# LncRNA LINC01094 Promotes Cells Proliferation and Metastasis through the PTEN/AKT Pathway by Targeting AZGP1 in Gastric Cancer

**DOI:** 10.3390/cancers15041261

**Published:** 2023-02-16

**Authors:** Zhe Gong, Yanqiu Zhang, Yue Yang, Yanan Yang, Jieyun Zhang, Yixuan Wang, Liqin Zhao, Nuoya Yu, Zhenhua Wu, Weijian Guo

**Affiliations:** 1 Department of Gastrointestinal Medical Oncology, Fudan University Shanghai Cancer Center, Shanghai 200032, China; 2 Department of Oncology, Shanghai Medical College, Fudan University, Shanghai 200032, China; 3 Department of Radiation Oncology, Shandong Cancer Hospital and Institute, Shandong First Medical University and Shandong Academy of Medical Sciences, Jinan 250000, China; 4 Department of Oncology, Ruijin Hospital, Shanghai Jiao Tong University School of Medicine, Shanghai 200025, China

**Keywords:** lncRNA LINC01094, AZGP1, metastasis, prognosis, gastric cancer

## Abstract

**Simple Summary:**

Long noncoding RNAs (lncRNAs) were recently reported to play an essential role in multiple cancer types. We identified a lncRNA LINC01094, which was associated with the metastasis of GC through next-generation sequencing. The high expression of LINC01094 was associated with high T and N stages and a poor prognosis. We found that LINC01094 promotes the proliferation and metastasis of GC in vitro and in vivo. AZGP1 was found as the protein-binding partner of LINC01094 by using RNA pulldown and RNA-binding protein immunoprecipitation (RIP) assays. LINC01094 antagonizes the function of AZGP1, downregulates the expression of PTEN, and further upregulates the AKT pathway. Collectively, our results suggested that LINC01094 might predict the prognosis of GC patients and become the therapy target for GC.

**Abstract:**

Long noncoding RNAs (lncRNAs) were recently reported to play an essential role in multiple cancer types. Herein, through next-generation sequencing, we screened metastasis-driving molecules by using tissues from early-stage gastric cancer (GC) patients with lymph node metastasis, and we identified a lncRNA LINC01094, which was associated with the metastasis of GC. According to the clinical data from the TCGA, GSE15459, and GSE62254 cohorts, the high expression of LINC01094 was associated with an unfavorable prognosis. Moreover, 106 clinical GC and paired normal samples were collected, and the qRT-PCR results showed that the high expression of LINC01094 was associated with high T and N stages and a poor prognosis. We found that LINC01094 promotes the proliferation and metastasis of GC in vitro and in vivo. AZGP1 was found as the protein-binding partner of LINC01094 by using RNA pulldown and RNA-binding protein immunoprecipitation (RIP) assays. LINC01094 antagonizes the function of AZGP1, downregulates the expression of PTEN, and further upregulates the AKT pathway. Collectively, our results suggested that LINC01094 might predict the prognosis of GC patients and become the therapy target for GC.

## 1. Introduction

GC earned the fifth highest rate of incidence and the fourth leading cause of cancer-related deaths among all tumors worldwide [1]. GC is insidious in onset, so most patients are diagnosed with metastatic GC. Usually, GC patients with metastases have a poorer prognosis [2,3,4]. Thus, it is an urgent to study the underlying mechanism that affects GC metastasis.

Over the past several years, some studies [5,6] have revealed that tumor metastasis is an early event in the process of tumor development. Thus, it is an urgent to find the key molecule that affects the process of tumor metastasis. Through early intervention, we might improve the outcomes for cancer patients.

In recent years, the role of lncRNAs in carcinogenesis and tumor development has received increasing attention. In the field of GC, researchers have found that a variety of lncRNAs have the potential to be used as biomarkers for the early screening, early diagnosis, prognosis prediction, and invasive ability prediction of GC [7,8]. For instance, H19 may be used for the early screening of GC [9], LINC00152 may be used to detect GC and correlate with the invasive ability of GC [10], and UCA1 may be used for the early diagnosis and prognosis prediction of GC [11]. These studies all suggest that lncRNAs play an important role in the occurrence and development of GC and may become therapeutic targets.

Many metastasis-related genes have been found in GC, but the mechanism of GC metastasis has not yet been elucidated. The exploration of lncRNAs brings opportunities for the elucidation of GC metastasis mechanisms, but it is still in its infancy. High-throughput sequencing technology brings convenience to the screening of lncRNAs related to tumor metastasis, while it is a challenge to screen from the massive data which driver molecules play important roles. Therefore, we utilized next-generation sequencing to perform a comparative study of early-stage GC patients with lymph node metastasis (high metastasis tendency) and GC patients with no metastasis (low metastasis tendency), combined with an analysis of public data set, to screen lncRNAs that play an important part in regulating GC metastasis. We hope that through this study, we could provide a novel insight in prognosis predicating and treatment strategies of GC.

## 2. Materials and Methods

### 2.1. Patients and Sample Collection

In total, 140 gastric cancer and paired normal tissues were obtained from the Biobank in Fudan University Shanghai Cancer Center (FUSCC). Among the 140 paired tissues, 34 paired tissues were used for next-generation sequencing, while 106 paired tissues were used to isolate RNA and qRT-PCR assays. All patients were enrolled at FUSCC from 2007 to 2015 in China. All patients underwent gastrectomy and were pathologically diagnosed as gastric adenocarcinoma. The clinical medical information was obtained from the medical records of FUSCC. The overall survival (OS) time was defined as the time from the date of surgery to the date of death or to the last follow-up. Written informed consent was obtained from all participants for their tissues to be utilized, and the application of the patient tissue sample and the study was approved by the FUSCC ethics committee.

As for public databases, 279, 192, and 299 GC patients with available lncRNA sequencing and OS data form the TCGA, GSE15459, and GSE62254 data sets, respectively, were also included in our study.

### 2.2. Total RNA Isolation, Reverse Transcription, and qRT-PCR

By following standard procedures, total RNA was isolated using TRIzol (Invitrogen, Carlsbad, CA, USA), and cDNA was synthesized using the PrimeScript RT Reagent Kit (Takara, Shiga, Japan). QRT-PCR was carried out on Mastercycler EP Realplex (Eppendorf, Hamburg, Germany) using TB Green Premix Ex Taq II (Takara, Shiga, Japan). The primers were designed and synthesized by Sangon Biotech (Shanghai, China). All primers used in our study were listed in Table S1.

### 2.3. Subcellular Fractionation

The PARIS Kit (Thermo Fisher, Waltham, MA, USA) was used to perform nuclear/cytoplasmic fractionation. According to the manufacturers’ instructions, 1 × 10^7^ HGC-27 or MGC-803 cells were suspended in cell fractionation buffer. Next, RNA was isolated, and qRT-PCR was performed. GAPDH and U1 small nuclear RNA served as the cytoplasmic and the nuclear endogenous controls, respectively.

### 2.4. RNA Fluorescence In Situ Hybridization (FISH)

LINC01094-fluorescence conjugated probes were designed and purchased from RiboBio (Guangzhou, China). By following standard procedures, the RNA FISH assay was conducted using the Fluorescent In Situ Hybridization Kit (Ribo). The HGC-27 or MGC-803 cells were counterstained with DAPI, and images were acquired by using a confocal microscope (LEICA SP5, Leica Microsystems, Wetzlar, Germany).

### 2.5. Next-Generation Transcriptome Sequencing

The total RNAs from tissues were treated with Ribo-off rRNA Depletion Kit (Vazyme, Nanjing, China). Detailed sequencing processes were described previously [6]. Sequencing reads from RNA-seq data were mapped to the human reference genome (GRCH38/hg38) through HISAT2. Gene-expression levels were calculated by using the FPKM (fragments per kilobase of transcript per million mapped reads).

### 2.6. Cell Lines and Culture Conditions

The human gastric cancer cell lines HGC-27 (RRID: CVCL_1279), MGC-803 (RRID: CVCL_5334), AGS (RRID: CVCL_0139), and MKN-28 (RRID: CVCL_1416) and the human gastric normal epithelial cell line GES-1 (RRID: CVCL_EQ22) were purchased from the cell bank of the Chinese Academy of Science (Shanghai, China). All cell lines have been authenticated by using STR profiling, in 2019. All cells were maintained in DMEM medium (Gibco, Carlsbad, CA, USA) supplemented with 10% fetal bovine serum (FBS, Gibco, Carlsbad, CA, USA) at 37 °C in a humidified atmosphere with 5% CO_2_. All experiments were performed with mycoplasma-free cells.

### 2.7. Short Tandem Repeat (STR) Profiling for Cell Lines

By following standard procedures, genomic DNA was extracted with the genomic DNA purification kit (Thermo Fisher, Waltham, MA, USA) and amplified with 21-STR amplification scheme, and the STR site and the Amelogenin gender determining marker were detected on ABI 3730XL genetic analyzer (Thermo Fisher, Waltham, MA, USA).

### 2.8. Western Blot

The CD24, SNAIL, SLUG, E-cadherin, PTEN, phosphorylated AKT, total AKT, ACTIN, and GAPDH primary antibodies were purchased from Cell Signaling Technology (1:1000, Beverly, MA, USA). The AZGP1, histone H4, and PRP primary antibodies were purchased from Abcam (1:1000, Cambridge, UK). Following standard procedures, we lysed cells using RIPA buffer (Beyotime, Nantong, China) with protease inhibitors and phosphatase inhibitors (Bimake, Houston, TX, USA). The protein concentrations of the cell lysates were quantified by using the BCA protein assay kit (Thermo Fisher, Waltham, MA, USA). Finally, 20 mg protein was separated by 10% SDS-PAGE and then transferred into polyvinylidene fluoride (PVDF) membranes. The membranes were blocked with 10% skim milk for 1 h at room temperature and then probed with the primary antibodies over night at 4 °C. Next, the membranes were washed with TBST and incubated with the secondary antibodies, which were conjugated with horseradish peroxidase (HRP) for 1 h at room temperature. A luminescent image analyzer (ImageQuant LAS4000 mini) was utilized to detect the protein. All these experiments were performed in triplicate. Original blots can be found at supplementary File S1.

### 2.9. Transfection and Lentivirus Infection

The LINC01094 antisense oligonucleotide (ASO) and the corresponding negative control (NC) ASO were purchased from RiboBio (Guangzhou, China). The AZGP1 and PTEN overexpression plasmid and the corresponding NC plasmids were purchased from Hanyin Biotechnology (Shanghai, China). The AZGP1 and PTEN overexpression plasmid were constructed in the pcDNA3.1 vector. According to the manufacturer’s instructions, the ASOs and plasmids were transfected into the cells using Lipofectamine 3000 for transient transfection. The LINC01094 overexpression lentiviral package was completed by Hanyin Biotechnology (Shanghai, China). The HGC-27 and MGC-803 cells were infected with LINC01094 overexpression lentivirus. The stable LINC01094 overexpression cells were selected by puromycin.

### 2.10. Transwell Migration Assay

In total, 4 × 10^4^ HGC-27 or MGC-803 cells in 200 μL serum-free medium were plated in the upper migration chambers with a pore size of 8 μm. The upper migration chambers had the noncoated membrane, and 600 μL containing 20% FBS medium was added into the lower migration chambers. All cells were maintained at 37 °C in a humidified atmosphere with 5% CO_2_. After incubation for 24 h, the cells that invaded the lower side of the migration chambers were fixed with 4% paraformaldehyde, stained with 1% crystal violet, and counted under an optical microscope (IX71, Olympus Life Science, Tokyo, Japan). All these experiments were performed in triplicate.

### 2.11. Proliferation and Colony-Formation Assays

For proliferation assay, 2000 HGC-27 or MGC-803 cells were seeded in a 96-well plate. All cells were maintained at 37 °C in a humidified atmosphere with 5% CO_2_. According to the manufacturer’s instructions, cell proliferation was assessed with cell counting kit-8 (CCK-8, Dojindo, Kumamoto, Japan). Cells were measured continuously for 5 days.

For colony-formation assay, 1000 HGC-27 or MGC-803 cells were seeded in a 6-well plate. All cells were maintained at 37 °C in a humidified atmosphere with 5% CO_2_. After incubation for about 2 weeks, visible colonies were fixed with 4% paraformaldehyde, stained with 1% crystal violet, and counted. All these experiments were performed in triplicate.

### 2.12. In Vitro Sphere-Forming Assay

A total of 500 mL sphere-forming media system contained DMEM (Gibco, Carlsbad, CA, USA) 250 mL, DMEM-F12(Gibco, Carlsbad, CA, USA) 250 mL, serum-free B27 (50×, Invitrogen, Carlsbad, CA, USA) 2 ml, EGF (Pepro Tech, Rocky Hill, NJ, USA) 5 ng, bFGF (Pepro Tech, Rocky Hill, NJ, USA) 5 ng, and BSA (Sigma, St.Louis, MO, USA) 2 g. In total, 4500 HGC-27 or MGC-803 cells in 500 μL sphere-forming media system were seeded in ultralow attachment 24-well plate (Corning, Corning, NY, USA). All cells were maintained at 37 °C in a humidified atmosphere with 5% CO_2_. Cells were cultured for 1–2 weeks to allow single cells to form spheres. After 1–2 weeks, the spheres were observed under an optical microscope (IX71, Olympus Life Science, Tokyo, Japan). All these experiments were performed in triplicate.

### 2.13. Transplantable Xenograft Mouse Model

In total, 24 male BALB/c nude mice aged 4 weeks, weighing 18–22 g, were provided by FUSCC laboratory animal science center and raised in a specific pathogen-free environment. For the subcutaneous tumor model, 1 × 10^7^ treated MGC-803 cells suspended in 200 μL of PBS were subcutaneously injected in the right forelimb of the mice, 6 mice per group. After feeding for 7 weeks, the nude mice were euthanized and sacrificed with CO_2_. The tumor tissues were removed and weighted. In addition, the growth of the xenografted tumor was observed. For the metastasis model, 2 × 10^6^ treated MGC-803 cells suspended in 200 μL of PBS were intravenously injected into the tails of the nude mice, 6 mice per group. After feeding for 4 weeks, the nude mice were euthanized and sacrificed with CO_2_ for dissection. The lung metastatic loci were examined by HE staining under microscope (BX51M, Olympus Life Science, Tokyo, Japan). Five random slices from each mouse were used for metastasis area calculation by ImageJ software.

### 2.14. HE Staining

According to the standard protocols, after dewaxing, slices were incubated with hematoxylin for 2 min and thereafter stained with eosin. Dehydrated slices were sealed with neutral gum for further analysis.

### 2.15. Immunohistochemistry

The Ki-67 level of tumor tissues was detected via immunohistochemistry (IHC) stain. Formalin-fixed paraffin-embedded tissues were cut into 4 μm sections. Next, the sections were routinely dewaxed and immersed in 3% H_2_O_2_ to block endogenous peroxidase activity. To avoid nonspecific binding, the sections were dripped with 10% goat serum. The PCNA primary antibody (1:5000, Cell Signaling Technology, Beverly, MA, USA) incubation was conducted overnight at 4 °C. The sections were incubated with secondary antibody for 15 min at room temperature, developed via diaminobenzidine, and observed under a microscope (BX51M, Olympus Life Science, Tokyo, Japan).

### 2.16. RNA Pulldown Assay

The LINC01094 in vitro transcription plasmid was purchased from Hanyin Biotechnology (Shanghai, China). The LINC01094 in vitro transcription plasmid was constructed in the pcDNA3.1 vector. The T7 and SP6 RNA polymerase promoter sequences were added to the start and the end of the LINC01094 sequence, respectively. In vitro transcription of LINC01094 and that of its antisense chain were performed using MEGAscript T7 and SP6 transcription kits (Thermo Fisher, Waltham, MA, USA), respectively. Thereafter, LINC01094 and its antisense chain were biotin labeled by pierce RNA3′ end biotinylation kit (Thermo Fisher, Waltham, MA, USA). Pierce magnetic RNA-protein pulldown kit was utilized to perform pulldown assay, and the RNA-protein complex was analyzed by using mass spectrum and western blot.

### 2.17. Mass Spectrometry

The RNA-protein complex was separated using SDS-PAGE, followed by silver staining. After centrifugation and drying, trypsin was added and digested at 37 °C overnight. According to the standard protocols, the lysate was analyzed by Easy-nLC 1000 (Thermo Fisher, Waltham, MA, USA) and Q Exactive (Thermo Fisher, Waltham, MA, USA). The raw data were analyzed using Proteome Discoverer 1.4.

### 2.18. RNA-Binding Protein Immunoprecipitation (RIP) Assay

According to the manufacturer’s instructions, EZ-magna RIP RNA-binding protein immunoprecipitation kit (Millipore, Billerica, MA, USA) was utilized to perform RIP assay. A rabbit anti-AZGP1 antibody or a rabbit IgG was used in the RIP assay. The coprecipitation RNAs were detected by using qRT-PCR.

### 2.19. Statistical Analysis

All our analyses were conducted using GraphPad Prism 7.0 software (GraphPad Software, SanDiego, CA, USA), R software version 3.6.0 (https://www.r-project.org/, Accessed on 28 September 2020), and SPSS 20.0 (SPSS Inc., Chicago, IL, USA). Kaplan–Meier curve and Log-rank test was used to evaluate the relationship between different groups and overall survival (OS). Paired *t* test was used to compare variables between groups. Correlations between categorical variables were evaluated by using the chi-square and Kruskal–Wallis tests. Here, *p*-value < 0.05 was considered statistically significant.

## 3. Results

### 3.1. LINC01094 Was Correlated with Early Metastasis and Prognosis in GC

**Figure 1:**
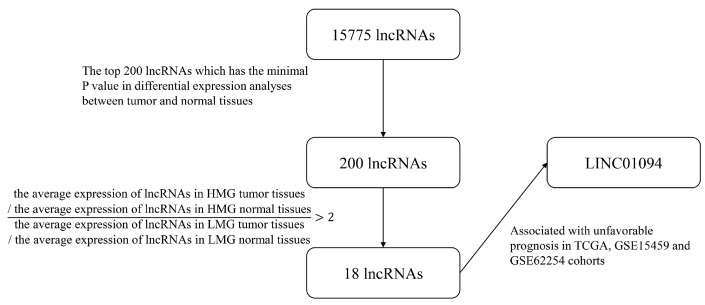
The workflow of the screening process of lncRNAs.

**Figure 2:**
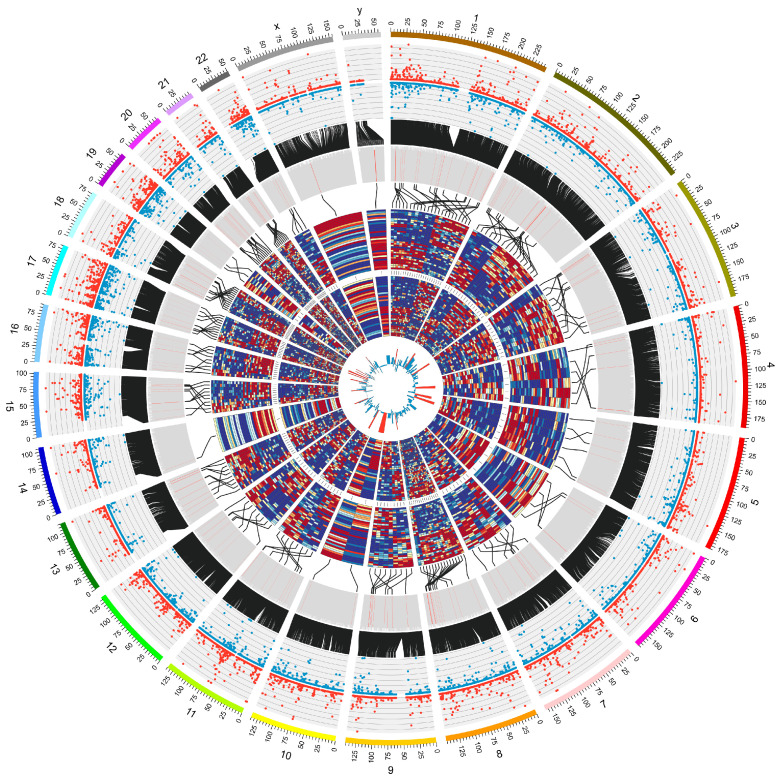
Circos plot displayed screening of metastasis-related lncRNAs in FUSCC RNA sequence data. Circos plot displayed the distribution and expression of lncRNAs on human chromosomes. The outermost layer was a chromosome map of the human genome. The inner scatter diagrams corresponded to the distribution and expression of detected lncRNAs on the chromosomes; the red represents tumor tissue, while the blue represents normal tissue. The next inner histogram corresponded to the *p*-value for the differential expression analysis between tumor tissue and normal tissue, and the first 200 *p*-values were marked red. The next inner heat map from outside to inside corresponded to the expression of the first 200 lncRNAs in tumor tissue of HMG, normal tissue of HMG, tumor tissue of LMG, and normal tissue of LMG. The innermost histogram corresponded the expression ratio between HMG and LMG; ratios > 2 were marked red; and LINC01094 was represented by the red column, pointing to chromosome 4.

**Figure 3:**
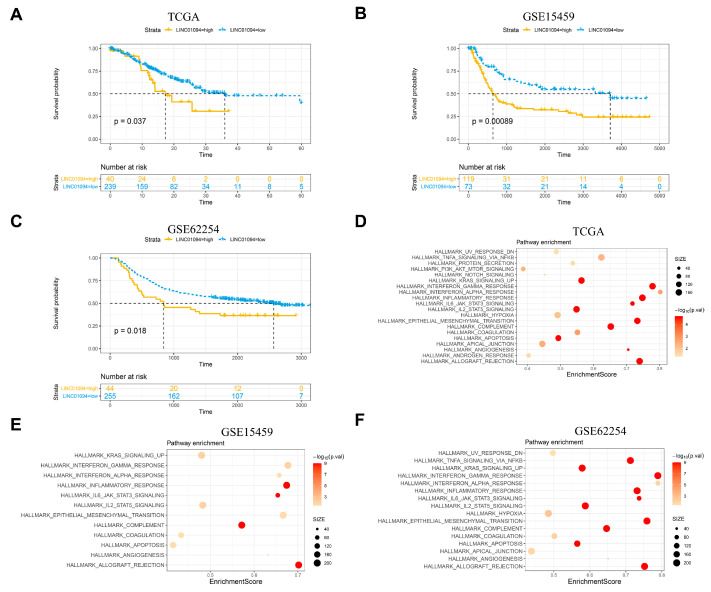
Survival and GSEA analysis in TCGA and GEO data sets. Kaplan–Meier survival plot showed that LINC01094 high-expression gastric cancer (separated by cutoff expression of LINC01094 calculated by ROC analyses) conferred a worse prognosis in the TCGA gastric cancer (**A**), GSE15459 (**B**), and GSE62254 (**C**) cohorts. Upregulated pathways analyzed by GSEA in TCGA gastric cancer cohort (**D**), GSE15459 (**E**), and GSE62254 (**F**).

**Figure 4:**
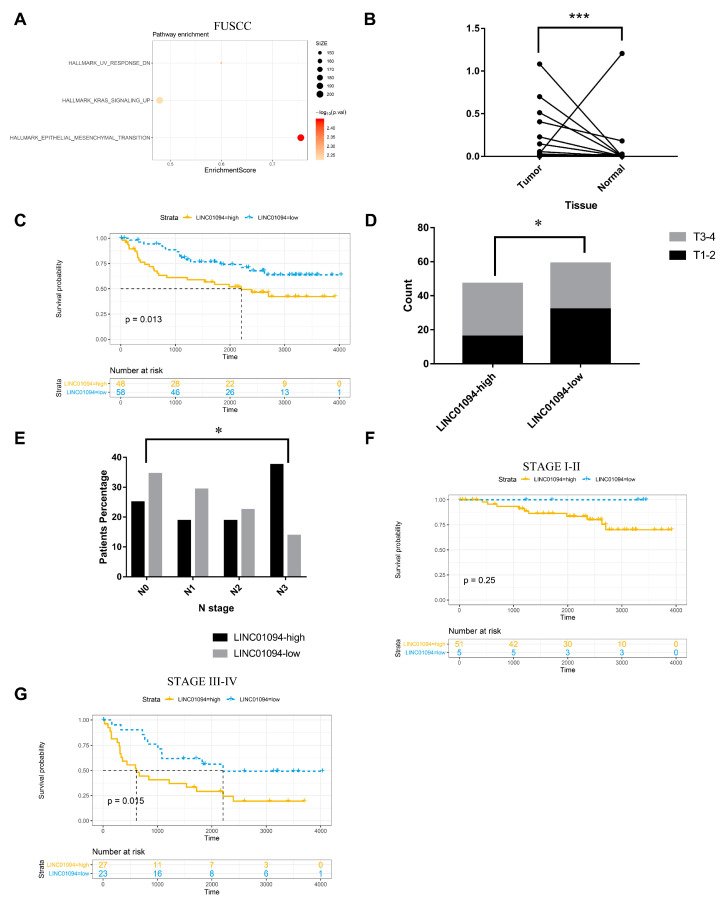
Analysis in FUSCC RNA sequence and PCR data. (**A**) Upregulated pathways analyzed by GSEA in FUSCC RNA sequence data. (**B**) LINC01094 expression was evaluated in 106 paired cancerous and noncancerous tissues from FUSCC. (**C**) For the whole cohort, the Kaplan–Meier curve showed that LINC01094 high expression was associated with an unfavorable prognosis. Patients in the LINC01094 high-expression group had higher T (**D**) and N (**E**) stages. (**F**) For the stage I–II subgroup, the Kaplan–Meier curve showed that LINC01094 high expression was associated with an unfavorable prognosis. (**G**) For the stage III–IV subgroup, the Kaplan–Meier curve showed that LINC01094 high expression was associated with an unfavorable prognosis. *: *p* < 0.01, ***: *p* < 0.001.

To uncover the novel lncRNAs that correlated with early metastasis in GC, next-generation transcriptome sequencing was used to detect the lncRNA expression profile of GC tissues, and it matched normal tissues from 17 GC patients in the T1 and N1–3 stages (defined as high metastatic tendency group (HMG)) and 17 GC patients in any T or N0 stage (defined as low metastatic tendency group (LMG)). The workflow that illustrated the screening process of lncRNAs was demonstrated in Figure 1. A circos [12] figure was constructed to visualize the results of sequencing and the process of screening (Figure 2). In total, 15,775 lncRNAs were detected from NGS. An R package called “limma” was used to identify the differential expression profiles of lncRNA between 34 pairs of GC tissues (including HMG and LMG) to find the lncRNAs that had the biggest different expression between GC and paired normal tissues. The top 200 lncRNAs that had the minimal *p*-value were selected (Table S2). Next, 18 lncRNAs (Table S2) were further selected on the basis of an expression ratio (the ratio was calculated as follows: expression ratio = (the average expression of lncRNAs in HMG tumor tissues/the average expression of lncRNAs in HMG normal tissues)/(the average expression of lncRNAs in LMG tumor tissues/the average expression of lncRNAs in LMG normal tissues)) >2. For the 18 lncRNAs, we found that LINC01094 was associated with an unfavorable prognosis in the TCGA, GSE15459, and GSE62254 cohorts (Figure 3A–C). GSEA results showed that the upregulated EMT, KRAS-signaling, and stemness-related pathways, such as the IL6-JAK-STAT3- and IL2-STAT5-signaling pathways, were shown as upregulated in the LINC01094 high-expression group in the TCGA, GSE15459, and GSE62254 cohorts (Figure 3D–F, respectively). The upregulated EMT and KRAS-signaling pathways were also detected in the LINC01094 high-expression group in the FUSCC cohort (Figure 4A).

Thus, we further collected 106 gastric cancer and paired normal tissues from the Biobank in FUSCC to explore whether LINC01094 has the potential to be a marker for predicting metastasis and clinical prognosis in patients with gastric cancer. Patient characteristics are shown in Table 1. QRT-PCR was utilized to detect the expression of LINC01094. As is shown in Figure 4B, the expression of LINC01094 in tumor tissue was significantly higher than in normal tissue (*p* < 0.05). We used the tumor/normal tissue expression fold to divide the 106 patients into LINC01094 high-expression and low-expression groups, with the cutoff of 2.5 calculated by receiver operating characteristic (ROC) analyses. The Kaplan–Meier curve showed that LINC01094 high expression was associated with an unfavorable prognosis (Figure 4C, *p* < 0.05). Moreover, patients in the LINC01094 high-expression group had higher T and N stages (Figure 4D,E, all *p* < 0.05). In particular, the only patient with a distant metastasis belonged to the LINC01094 high-expression group, and they were diagnosed with N3. We also investigated the prognosis predictive value of LINC01094 in GC patients diagnosed with stage I–II or stage III–IV. For the stage I–II subgroup (N = 56), patients were divided into the LINC01094 high-expression and low-expression groups, with the cutoff of 0.11 calculated by ROC analyses, and the Kaplan–Meier curve showed that LINC01094 high expression was associated with an unfavorable prognosis, although the differences in OS between the LINC01094 high-expression and the LINC01094 low-expression groups did not show a statistically significant difference (Figure 4F, *p* = 0.25). In particular, no death event was recorded in the LINC01094 low-expression group. For the stage III–IV subgroup (N = 50), patients were divided into LINC01094 high-expression and low-expression groups, with the cutoff of 0.25 calculated by ROC analyses, and the Kaplan–Meier curve showed that LINC01094 high expression was associated with an unfavorable prognosis (Figure 4G, *p* < 0.05). Our results suggested that LINC01094 plays an important role in GC and is worth further exploration.

### 3.2. The Characteristics of LINC01094 in GC

**Figure 5:**
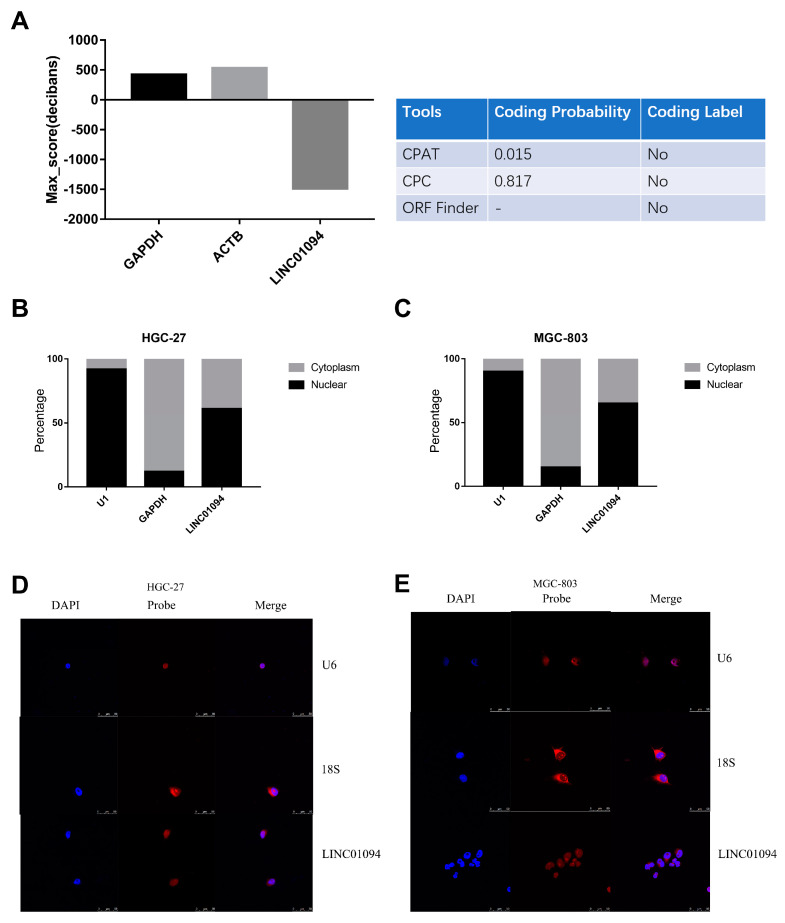
The characteristics of LINC01094 in GC. (**A**) LINC01094 was predicted as a noncoding RNA by PhyloCSF, CPAT, CPC, and the ORF finder. QRT-PCR for the separated nuclear and cytoplasmic fraction of HGC-27 (**B**) and MGC-803 (**C**) cell lines showed that LINC01094 was in both the nucleus and the cytoplasm. FISH assay showed that LINC01094 was in both the nucleus and the cytoplasm in the HGC-27 (**D**) and MGC-803 (**E**) cell lines (objective: 63×). The scale bar equals 50 μm, as indicated in each graph.

LINC01094 was in 4q21.21, and it was predicted as a noncoding RNA by PhyloCSF, CPAT, CPC, and the ORF finder (Figure 5A). Furthermore, qRT-PCR for the separated nuclear and cytoplasmic fraction of GC cell lines (Figure 5B,C) and FISH in GC cell lines showed that LINC01094 was in both the nucleus and the cytoplasm (Figure 5D,E).

### 3.3. LINC01094 Promotes Cells Proliferation and Metastasis in GC

**Figure 6:**
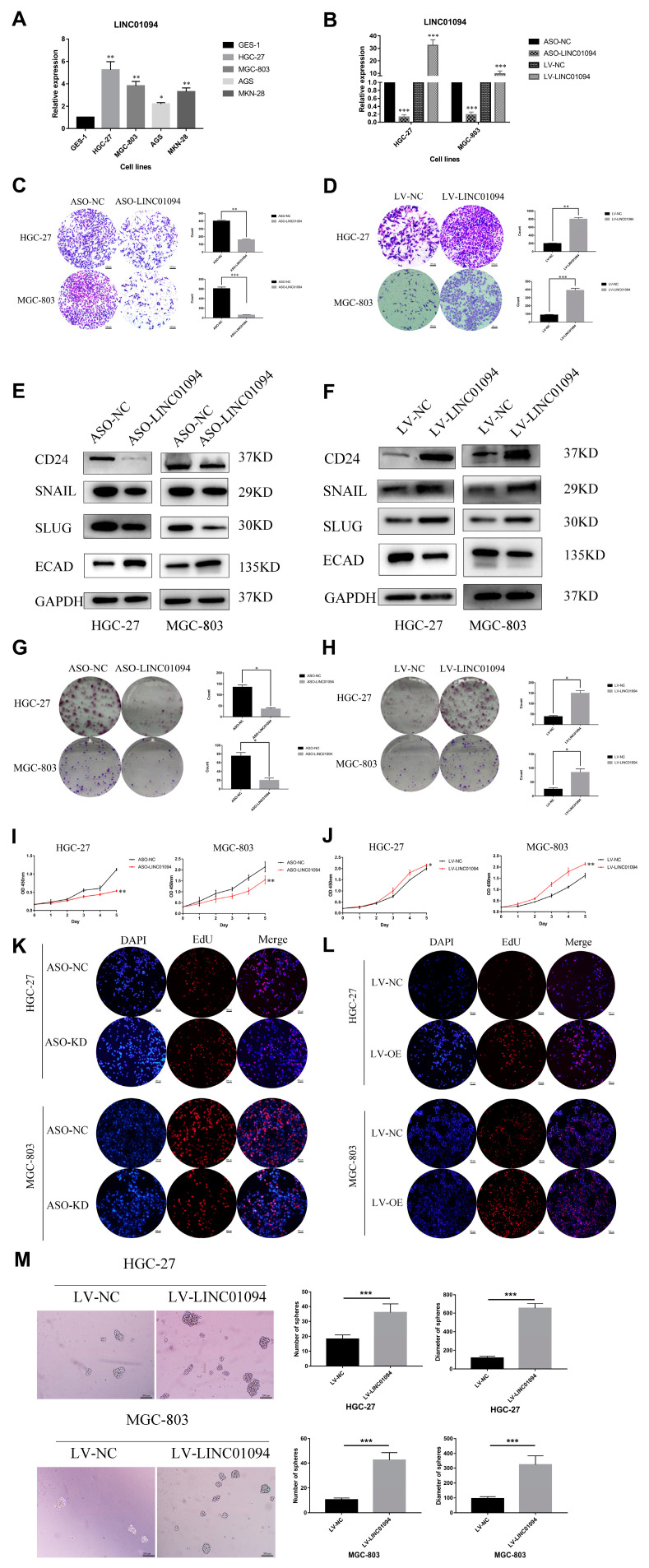
LINC01094 promotes the malignant phenotype of GC in vitro. (**A**) LINC01094 expression levels in the normal human gastric mucosal cell line (GES-1) and human gastric cancer cell lines. (**B**) QRT-PCR results showed the knockdown efficacy of ASOs and overexpression efficacy of lentivirus. The migration abilities were weakened after the knockdown of LINC01094 (**C**) and were strengthened after the overexpression of LINC01094 (**D**) (objective: 10×). Western blotting was applied to assess the expression levels of EMT and stemness-associated proteins after silencing (**E**) and overexpressing (**F**) LINC01094 in HGC-27 and MGC-803 cells, respectively. The colony-formation results showed that the proliferation of HGC-27 and MGC-803 cells was inhibited and strengthened after silencing (**G**) and overexpressing (**H**) LINC01094, respectively. (**I**) The CCK-8 assay showed that silencing LINC01094 weakened the proliferation of HGC-27 and MGC-803 cells. (**J**) The CCK-8 assay showed that overexpressing LINC01094 strengthened the proliferation of HGC-27 and MGC-803 cells. (**K**) The EdU assay showed that silencing LINC01094 weakened the proliferation of HGC-27 and MGC-803 cells (objective: 4×). (**L**) The EdU assay showed that overexpressing LINC01094 strengthened the proliferation of HGC-27 and MGC-803 cells (objective: 4×). (**M**) Sphere-forming assay showed that LINC01094 overexpression cell lines formed more spheres (objective: 4×). *: *p* < 0.01, **: *p* < 0.01, ***: *p* < 0.001. The scale bar equals 100 or 200 μm, as indicated in each graph.

The QRT-PCR results showed that LINC01094 had a relatively higher expression in GC cell lines (HGC-27, MGC-803, AGS, and MKN-28) than in the normal gastric mucosal epithelial cell line (GES-1) (Figure 6A). On the basis of the base expression level of LINC01094 (Figure 6A), we used GC cell lines HGC-27 and MGC-803 to explore the unction and underlying mechanism of LINC01094 in GC. The knockdown efficacy of ASOs and the overexpression efficacy of lentivirus were detected by using qRT-PCR (Figure 6B). The transwell assay was utilized to assess the effect of LINC01094 on migration. As is shown in Figure 6C,D, the migration abilities were weakened after the knockdown of LINC01094 and were strengthened after the overexpression of LINC01094. Our western blot results showed that LINC01094 might regulate the process of EMT. As is shown in Figure 6E, snail and slug were downregulated, whereas E-cadherin was upregulated after silencing LINC01094. Moreover, when overexpressing LINC01094, snail and slug were upregulated, whereas E-cadherin was downregulated (Figure 6F).

The colony-formation results showed that the proliferation of the HGC-27 and MGC-803 cells was inhibited and strengthened after silencing and overexpressing LINC01094, respectively (Figure 6G,H). Consistently, the CCK-8 assay and EdU assay also validated that silencing LINC01094 weakened the proliferation of HGC-27 and MGC-803 cells, while overexpressing LINC01094 strengthened the proliferation of HGC-27 and MGC-803 cells (Figure 6I–L).

Sphere-forming assays were utilized to explore the association between LINC01094 and the stemness of GC cells. As is shown in Figure 6M, LINC01094 overexpression cell lines formed more spheres. Furthermore, we also detected the expression of CD24, which was considered as a stemness-associated protein in LINC01094 overexpression cell lines and its negative control cell lines. The results showed that CD24 was downregulated after silencing LINC01094, while CD24 was upregulated after overexpressing LINC01094 (Figure 6E,F).

**Figure 7:**
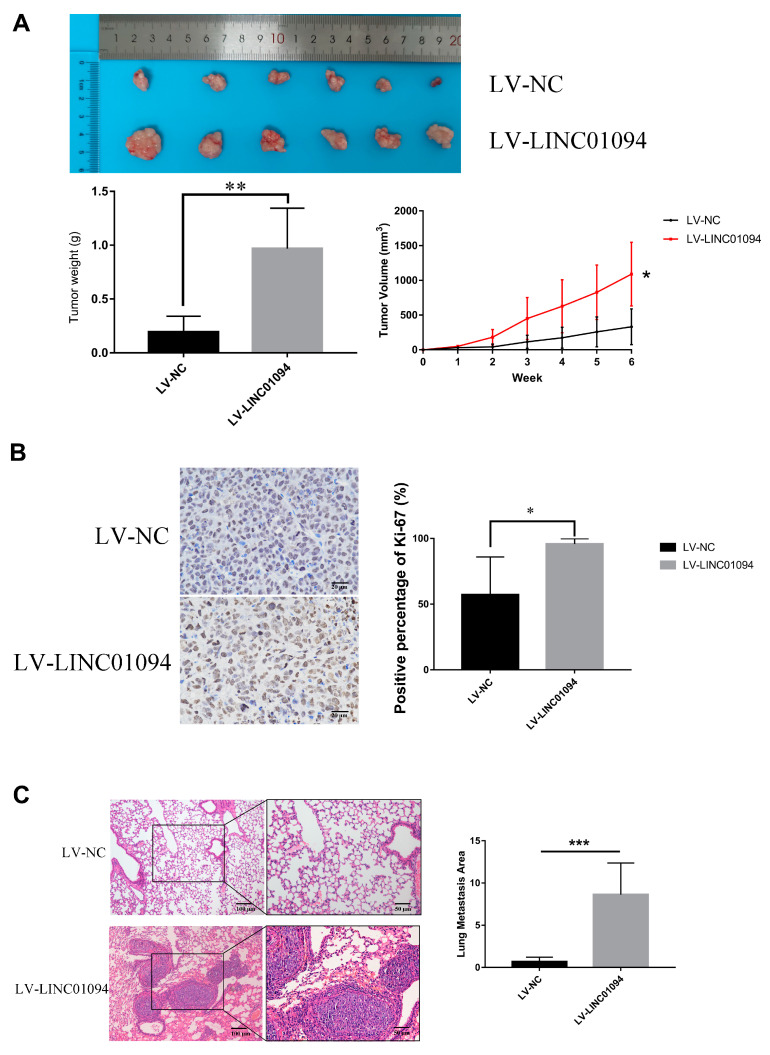
LINC01094 promotes the malignant phenotype of GC in vivo. (**A**) After overexpressing LINC01094, the proliferation of MGC-803 cells was strengthened in vivo. (**B**) Ki-67 IHC staining of tumor tissues dissected from subcutaneous xenograft models (objective: 20×). (**C**) The representative H- and E-stained slice pictures for the negative control and LINC01094 overexpression groups (objective: left 10×, right 20×). *: *p* < 0.01, **: *p* < 0.01, ***: *p* < 0.001. The scale bar equals 20, 50, or 100 μm, as indicated in each graph.

We also investigated the function of LINC01094 on GC growth and metastasis in vivo. As is shown in Figure 7A, after overexpressing LINC01094, the proliferation of the MGC-803 cells was strengthened in vivo. The IHC staining of Ki-67 verified the effect of LINC01094 on GC growth (Figure 7B). As for the metastasis model, the area of lung metastasis in the LINC01094 overexpression group was significantly larger than that of the NC group. Figure 7C showed the representative HE-stained slice pictures and the comparison of the area of lung metastasis for the NC and LINC01094 overexpression groups.

### 3.4. LINC01094 Directly Binds to AZGP1 in GC

**Figure 8:**
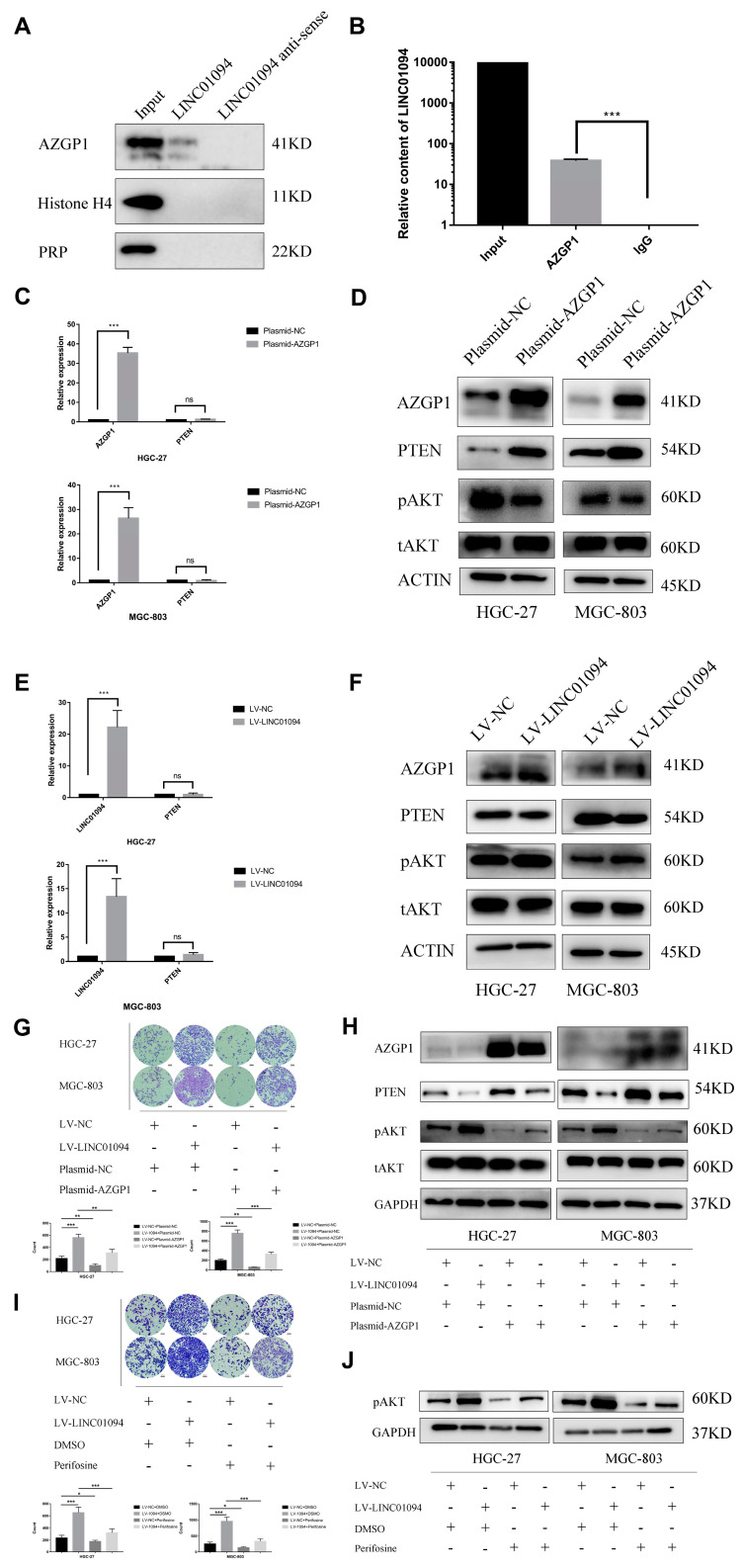
LINC01094 promotes migration of tumor cells through the PTEN/AKT pathway by targeting AZGP1 in GC. (**A**) The western blot results showed that only AZGP1 specifically combined LINC01094. (**B**) The qRT-PCR results showed that LINC01094 was significantly more enriched with the anti-AZGP1 antibody than with the rabbit IgG. (**C**) The qRT-PCR results showed that the RNA level of PTEN underwent no significant change after overexpression of AZGP1 in HGC-27 and MGC-803 cells. (**D**) Western blotting was applied to assess the expression levels of PTEN, pAKT, and total AKT after overexpressing AZGP1 in HGC-27 and MGC-803 cells. (**E**) The qRT-PCR results showed that the RNA level of PTEN underwent no significant change after overexpression of LINC01094 in HGC-27 and MGC-803 cells. (**F**) Western blotting was applied to assess the expression levels of AZGP1, PTEN, pAKT, and total AKT after overexpressing LINC01094 in HGC-27 and MGC-803 cells. (**G**) Rescue experiments showed that the migration-promotion effect caused by overexpression of LINC01094 could be rescued by overexpression of AZGP1 (objective: 10×). (**H**) The western blot results showed that the downregulation of PTEN and upregulation of pAKT effects caused by overexpression of LINC01094 could be rescued by overexpression of AZGP1. (**I**) Rescue experiments showed that the migration-promotion effect caused by overexpression of LINC01094 could be rescued by perifosine (objective: 10×). (**J**) The western blot results showed that perifosine successfully downregulated the expression of pAKT. *: *p* < 0.01, **: *p* < 0.01, ***: *p* < 0.001, ns: no statistical significance. The scale bar equals 100 μm, as indicated in each graph.

To explore the underlying mechanism of LINC01094 in GC, we performed a RNA-protein pulldown assay, coupled with mass spectrometry, in HGC-27 cells. The mass spectrometry results are shown in Table S3. The in vitro transcription of LINC01094 and that of its antisense chain were performed, and we compared the different proteins that were combined by the two chains. We searched for proteins that were considered as RNA-binding proteins and detected more in the LINC01094 sense chains. Next, AZGP1, Histone H4, and PRP were selected for further validation. The western blot results are shown in Figure 8A, and only AZGP1 specifically combined with LINC01094. We further performed a RIP assay to validate the direct combination between LINC01094 and AZGP1. The qRT-PCR results showed that LINC01094 was significantly more enriched with the anti-AZGP1 antibody than with the rabbit IgG (Figure 8B). Taken together, our results reveal that LINC01094 directly binds to AZGP1 in GC.

### 3.5. LINC01094 Regulates PTEN Expression by Antagonizing the Function of AZGP1

It has been revealed that AZGP1 could upregulate the expression of PTEN and regulate the PTEN/AKT pathway to suppress tumor development. Thus, we hypothesized that LINC01094 could antagonize the function of AZGP1 and promote the malignant phenotype in GC. To validate our hypothesis, we first transfected HGC-27 and MGC-803 cells with the AZGP1 overexpression plasmid. As is shown in Figure 8C, the RNA level of PTEN underwent no significant change, while the protein level of PTEN was upregulated after the overexpression of AZGP1 (Figure 8D). Moreover, the protein level of phosphorylated AKT (pAKT) was downregulated (Figure 8D). We also performed qRT-PCR assays to detect the RNA level changes of PTEN in LINC01094 overexpression GC cell lines, and the results showed that the RNA level of PTEN underwent no significant change after the overexpression of LINC01094 (Figure 8E). Therefore, we performed western blot assays to detect the expression of AZGP1, PTEN, and pAKT in LINC01094 overexpression GC cell lines. The results revealed that the AZGP1 underwent no significant change; the expression of PTEN was downregulated; and the expression of pAKT was upregulated (Figure 8F). These results supposed that LINC01094 might regulate PTEN expression by antagonizing the function of AZGP1 and further regulating the PTEN/AKT pathway.

### 3.6. LINC01094 Promotes Migration of Tumor Cells through the PTEN/AKT Pathway by Targeting AZGP1 in GC

We further explored whether LINC01094 promotes the migration ability in GC depending on AZGP1 and the PTEN/AKT pathway. Rescue experiments were performed by using AZGP1 overexpression plasmid and AKT phosphorylation inhibitor perifosine in LINC01094 overexpression GC cell lines, separately. The results showed that the migration-promotion, downregulation of PTEN, and upregulation of pAKT effects caused by the overexpression of LINC01094 could be rescued by the overexpression of AZGP1 and perifosine (Figure 8G–J). Thus, our research showed that LINC01094 antagonizes AZGP1 and further downregulates the expression of PTEN. To sum it up, LINC01094 promotes the migration of tumor cells through the PTEN/AKT pathway by targeting AZGP1 in GC.

## 4. Discussion

Recent studies [13,14,15,16] have shown that lncRNAs play important roles in the occurrence and development of malignant tumors. However, how lncRNAs influence the process of tumor metastasis is still unclear. Several previous studies [5,6] have revealed that tumor metastasis is an early event in the process of tumor development. Patients diagnosed with metastatic GC usually accept systemic therapy rather than surgery. It is hard to acquire metastatic tumor tissues. Therefore, we performed NGS to screen metastasis-driving molecules by using tissues from early-stage GC patients with lymph node metastasis. The lncRNA expression profile of GC tissues and matched normal tissues were detected from 17 GC patients in the T1 and N1–3 stages (defined as HMG) and 17 GC patients in any T or N0 stage (defined as LMG). We finally selected the GC metastasis-associated lncRNA LINC01094 by comparing HMG and LMG. Utilizing the GC data from TCGA and GEO databases, we found that a high expression of LINC01094 is associated with an unfavorable prognosis. The GSEA results also showed that cancer-promoting pathways are upregulated in the TCGA, GSE15459, GSE62254, and FUSCC cohorts. Thus, we further enrolled 106 GC patients; the chi-square and Kruskal–Wallis tests showed that higher LINC01094 expression was associated with worse T and N stages, while the Kaplan–Meier curve showed that higher LINC01094 expression (tumor/normal tissue expression fold >2.5) was associated with a poorer prognosis. We also performed subgroup analyses in GC patients diagnosed with stage I–II and stage III–IV. For the stage III–IV subgroup, using 2.5 as the cutoff tumor/normal tissue expression fold, high LINC01094 expression was still associated with an unfavorable prognosis. For the stage I–II subgroup, using 0.11 as the cutoff tumor/normal tissue expression fold, the Kaplan–Meier curve showed that patients in the LINC01094 low-expression group had an extremely good prognosis (no death event was recorded in the LINC01094 low-expression group), although this might be due to the small sample size. The differences in OS between the high and low LINC01094 expression groups did not reach a statistically significant difference. Our findings indicated that stage I–II GC patients with an extremely low expression of LINC01094 (tumor/normal tissue expression fold <0.11) might not need postoperative adjuvant chemotherapy, which means that LINC01094 has a potential guiding value for clinical GC treatment, and LINC01094 is worthy of future large-sample-size studies to verify its clinical value. The high consistency among our NGS, the qRT-PCR data, and the public data imply that LINC01094 plays an important part in GC.

LINC01094, also known as CTEPHA1, has been associated with chronic thromboembolic pulmonary hypertension in a previous study [17]. Recent studies [18,19,20,21,22] have also pointed that LINC01094 promotes the malignant phenotype of kidney cancer, glioma, and ovarian cancer. However, few studies have focused on the relationship between LINC01094 and GC.

By knocking down and overexpressing LINC01094, our in vitro experiments found that LINC01094 promotes the migration and proliferation abilities of GC cells. A sphere-forming assay revealed that LINC01094 strengths the stemness of GC cells. The protein level changes after knocking down and overexpressing LINC01094 were consistent with our in vitro experiments results. Utilizing the transplantable xenograft mouse model, we found that LINC01094 promotes the proliferation and metastasis abilities of GC in vivo.

Recent studies [23,24,25,26,27,28,29] have revealed that AZGP1 could suppress the malignant phenotype in several cancers, including GC, colorectal cancer, prostate cancer, hepatocellular carcinoma, and so on. In this study, our RNA pulldown and RIP assays found that LINC01094 binds AZGP1. We further revealed that LINC01094 promotes the migration ability in GC, depending on the binding of AZGP1 by the rescue assay. Many researchers have indicated that AZGP1 suppressed the development of tumors by upregulating the expression of PTEN [25,28]. PTEN is a dual phosphatase with both protein and lipid phosphatase activities and is regarded as a tumor suppressor. Previous studies [30,31] have shown that the PTEN/AKT pathway plays an important part in GC development, and some important oncogenes and microRNAs also fulfill their function in GC, depending on the PTEN/AKT pathway. Our study also found that LINC01094 downregulates the expression of PTEN, while the expression level of AZGP1 undergoes no significant change. Thus, LINC01094 might fulfill the “protein sponge” function, which means that LINC01094 antagonizes the function of AZGP1 but does not affect the expression of AZGP1. These results partially explain the reasons why a high expression of LINC01094 has been associated with a poor prognosis. The classical function of PTEN antagonizes the AKT signaling pathway. According to the AKT phosphorylation inhibitor rescue assay, we found that LINC01094 promotes the migration ability in GC, also depending on the AKT pathway. The AKT pathway is a core pathway in the tumor biology for most cellular processes in cancer that are attributed to kinase-signaling networks [32]. The AKT pathway plays an important part in tumor growth [33,34], and recent studies have even revealed that the AKT pathway regulates the expression of CCL2 and affects the tumor immune microenvironment [35,36]. Taken together, these results indicate that LINC01094 inhibitors (such as ASOs) might be helpful for GC therapy.

Previous studies [25,28] have revealed that AZGP1 fulfills its tumor suppression function by regulating the PTEN/AKT pathway, but the mechanism is unclear. An interesting finding of our study is that AZGP1 downregulates the expression of PTEN at the protein level rather than at the RNA level. The results indicate that AZGP1 might affect protein degradation and stability and further downregulate PTEN. However, the more specific mechanism of LINC01094 or AZGP1 regulating the protein level of PTEN is still unclear and is worth exploration and further study.

## 5. Conclusions

In conclusion, our study suggested that LINC01094 promotes cells proliferation and metastasis through the PTEN/AKT pathway by targeting AZGP1 in GC. Further investigation into LINC01094 may contribute to finding novel survival predictive biomarkers and novel therapy targets for GC.

## Figures and Tables

**Table 1:** Clinical-pathological characteristics of the 106 enrolled patients from FUSCC.

Factors		LINC01094-High		LINC01094-Low		*p*-Value
		N = 48		N = 58		
		N	%	N	%	
Gender						0.4212
	Male	38	79.17	42	72.41	
	Female	10	20.83	16	27.59	
Age						0.6143
	≥60	28	58.33	31	53.45	
	<60	20	41.67	27	46.55	
T						0.1503
	1	12	25.00	27	46.55	
	2	4	8.33	4	6.90	
	3	1	2.08	1	1.72	
	4	31	64.58	26	44.83	
N						0.04274
	0	12	25.00	20	34.48	
	1	9	18.75	17	29.31	
	2	9	18.75	13	22.41	
	3	18	37.50	8	13.79	
M						0.2694
	0	47	97.92	58	100.00	
	1	1	2.08	0	0.00	
Histology						0.3803
	Adenocarcinoma	39	81.25	50	86.21	
	Mucinous adenocarcinoma	6	12.50	3	5.17	
	Signet-ring cell carcinoma	3	6.25	5	8.62	
Grade						0.2628
	1–2	11	22.92	19	32.76	
	3–4	37	77.08	39	67.24	
Vessel invasion						0.212
	Invasion	24	50.00	22	37.93	
	No invasion	24	50.00	36	62.07	
Neuroinvasion						0.009942
	Invasion	25	52.08	16	27.59	
	No invasion	23	47.92	42	72.41	

## Data Availability

The data sets generated and analyzed during the current study are available in the TCGA and GEO, TCGA: https://portal.gdc.cancer.gov, accessed on 28 September 2020; GEO: https://www.ncbi.nlm.nih.gov/geo, accessed on 28 September 2020. FUSCC cohort data used and analyzed during the current study are available from the corresponding author on reasonable request.

## Supplementary Materials

The following supporting information can be downloaded at https://www.mdpi.com/article/10.3390/cancers15041261/s1, Table S1: Primers used in this study; Table S2: The top 200 lncRNAs that had the biggest different expression between GC and paired normal tissues; Table S3: Mass spectrometry results; File S1: uncropped western blot images.
