# Supplementary material for: LncRNA LINC01094 Promotes Cells Proliferation and Metastasis through the PTEN/AKT Pathway by Targeting AZGP1 in Gastric Cancer
Source: Cancers (Basel). 2023 Feb 16;15(4):1261. doi: 10.3390/cancers15041261 (PMC9954187; doi:10.3390/cancers15041261)

Figure 6E Left

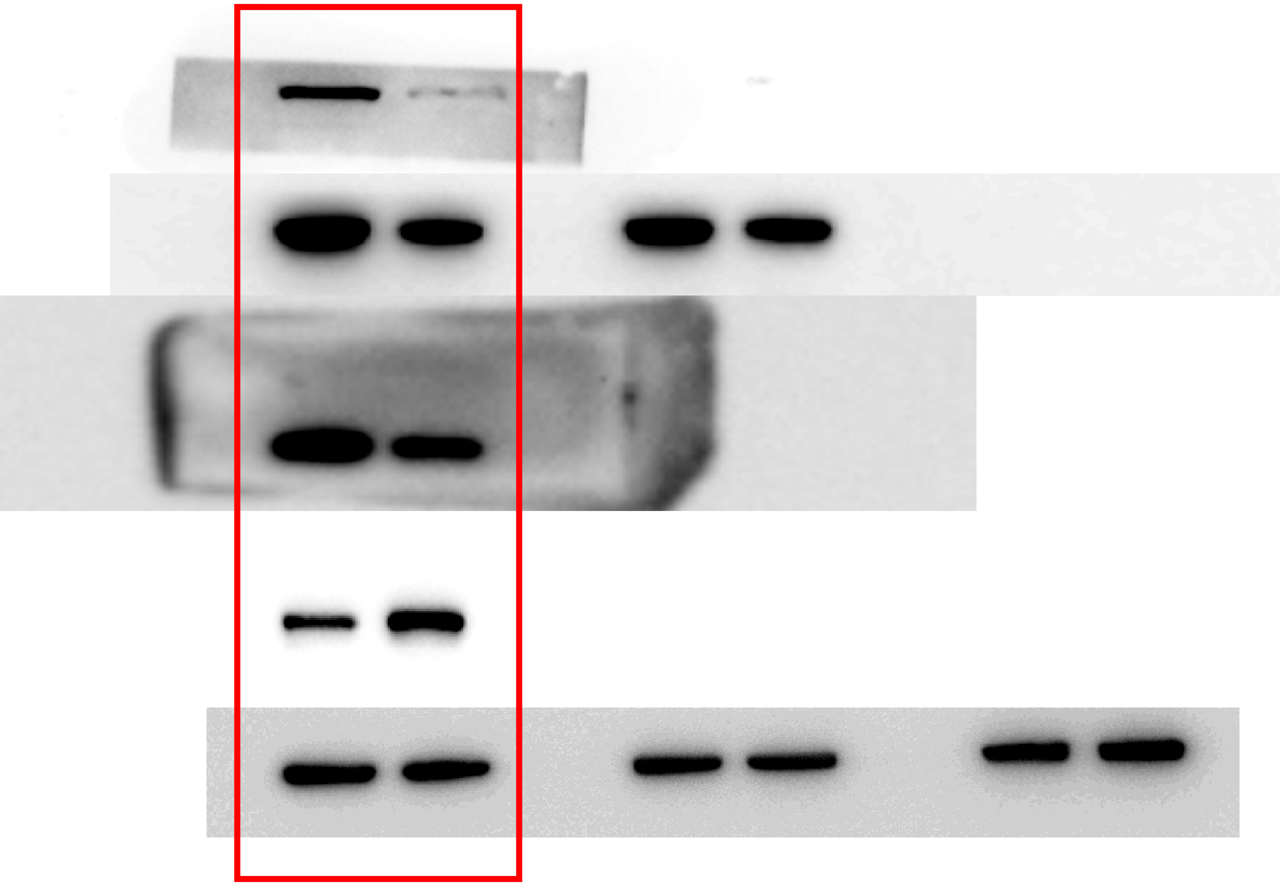

Figure 6E Right

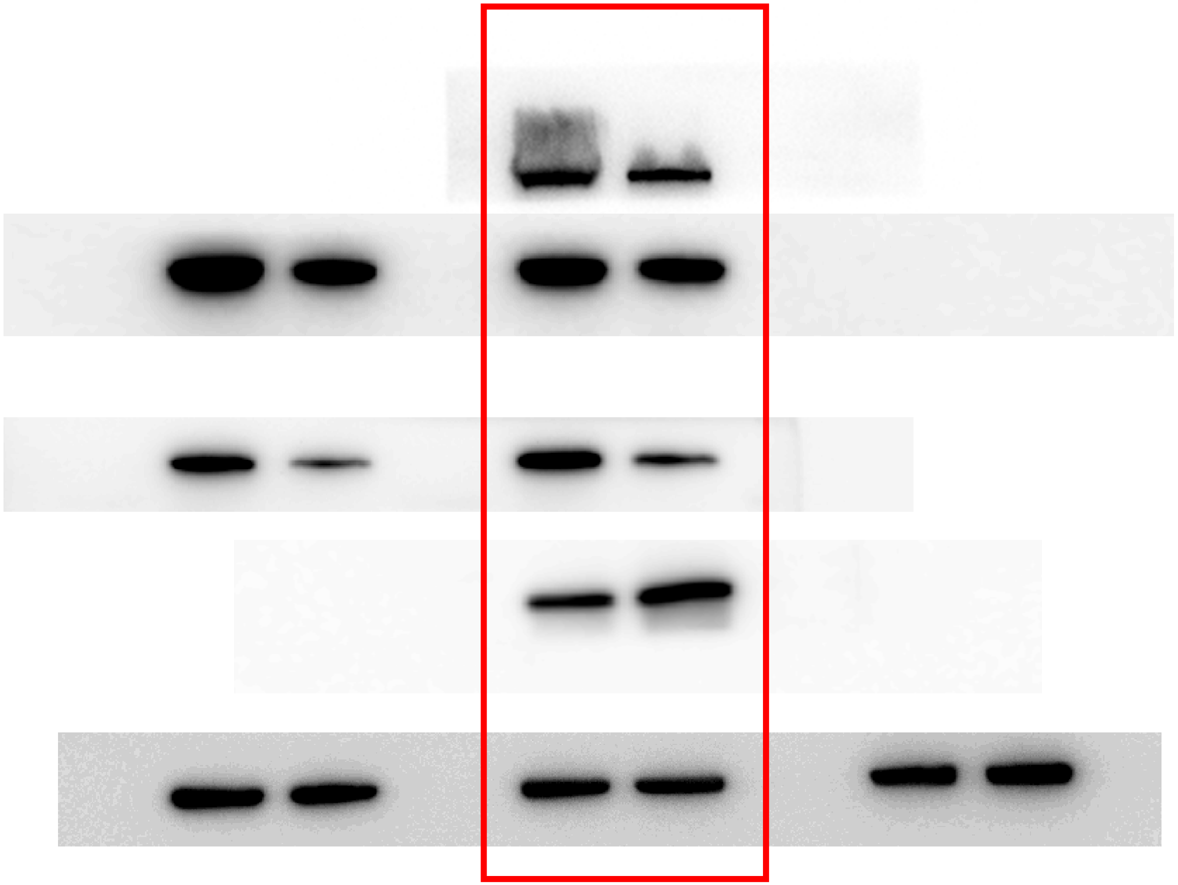

Figure 6F Left

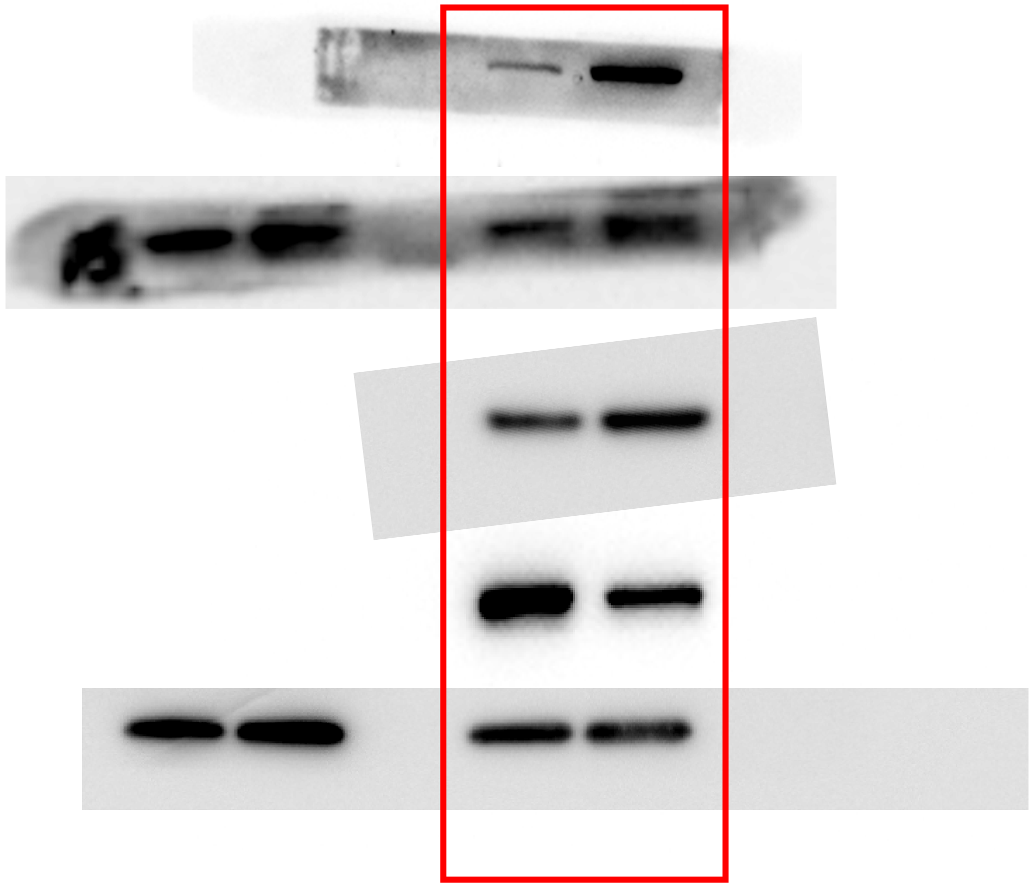

Figure 6F Right

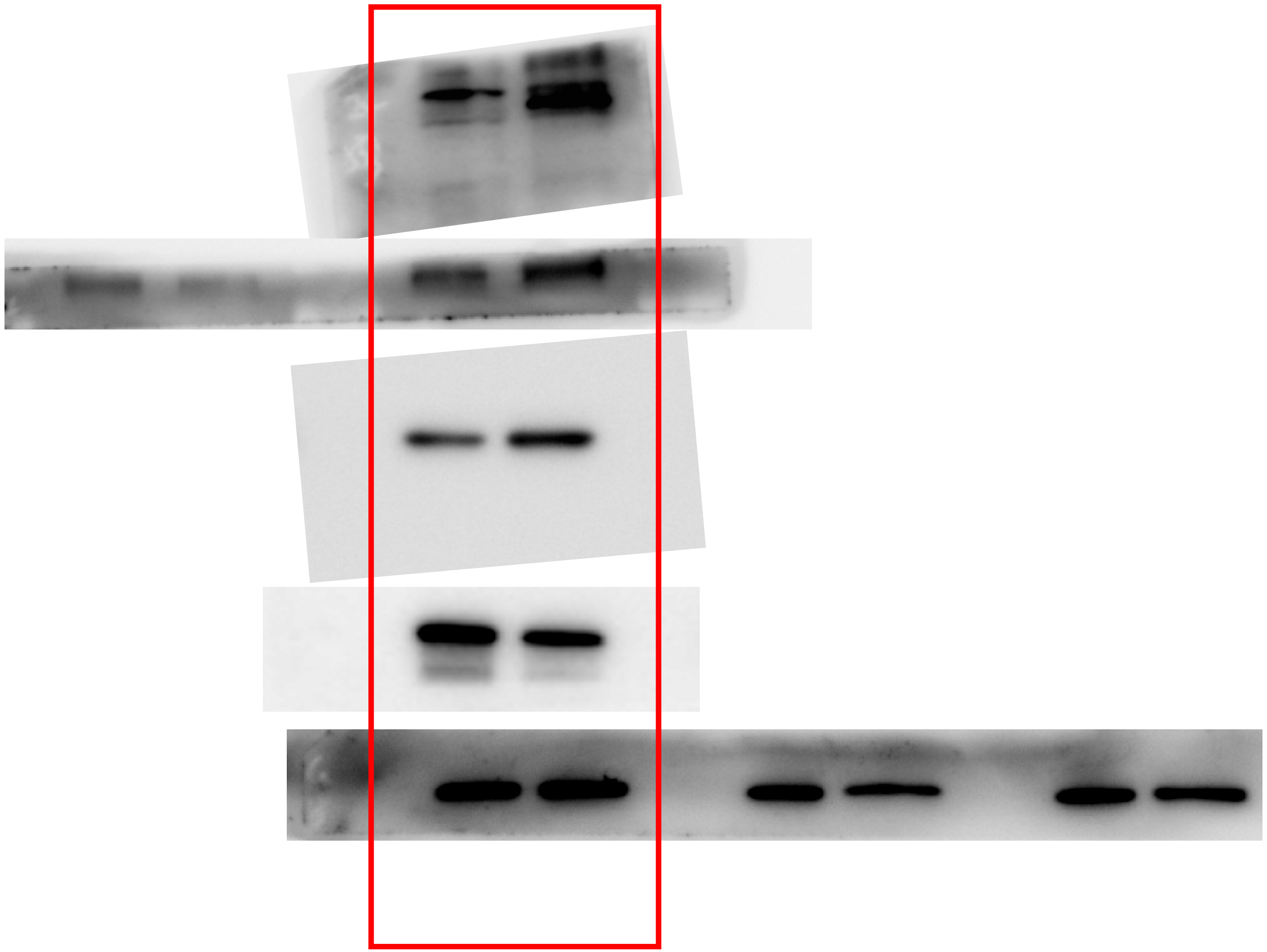

Figure 8A

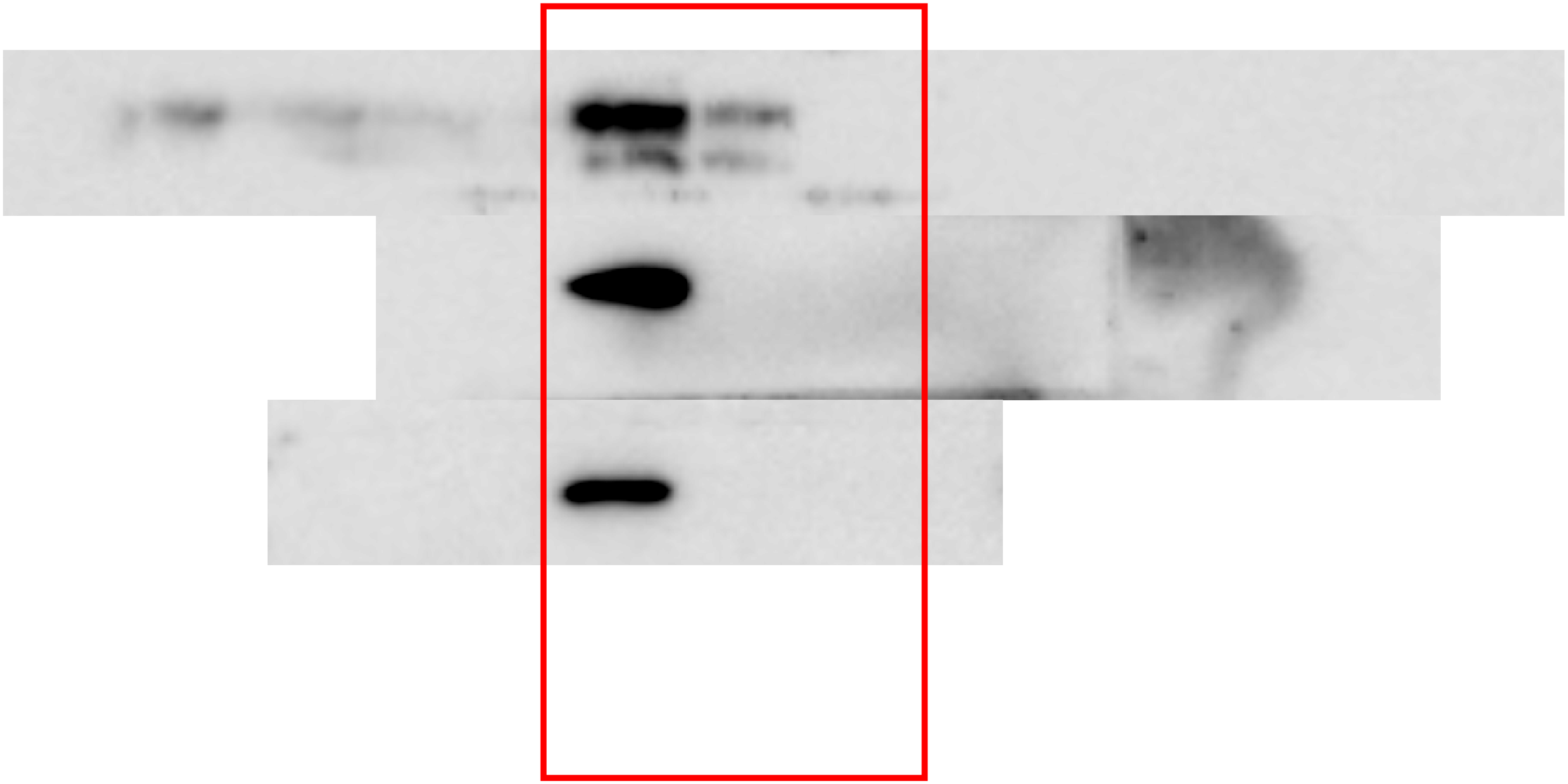

Figure 8D Left

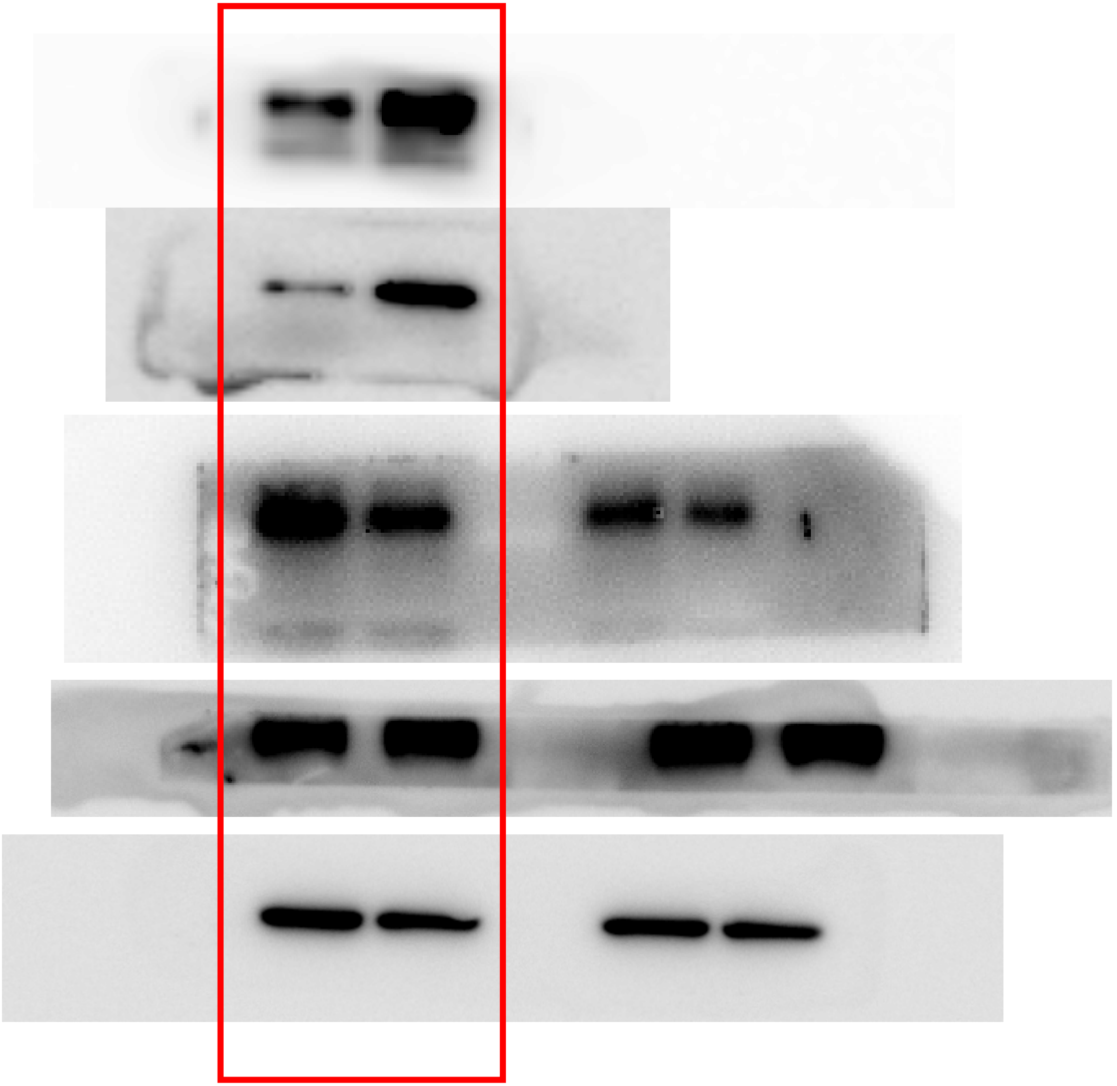

Figure 8D Right

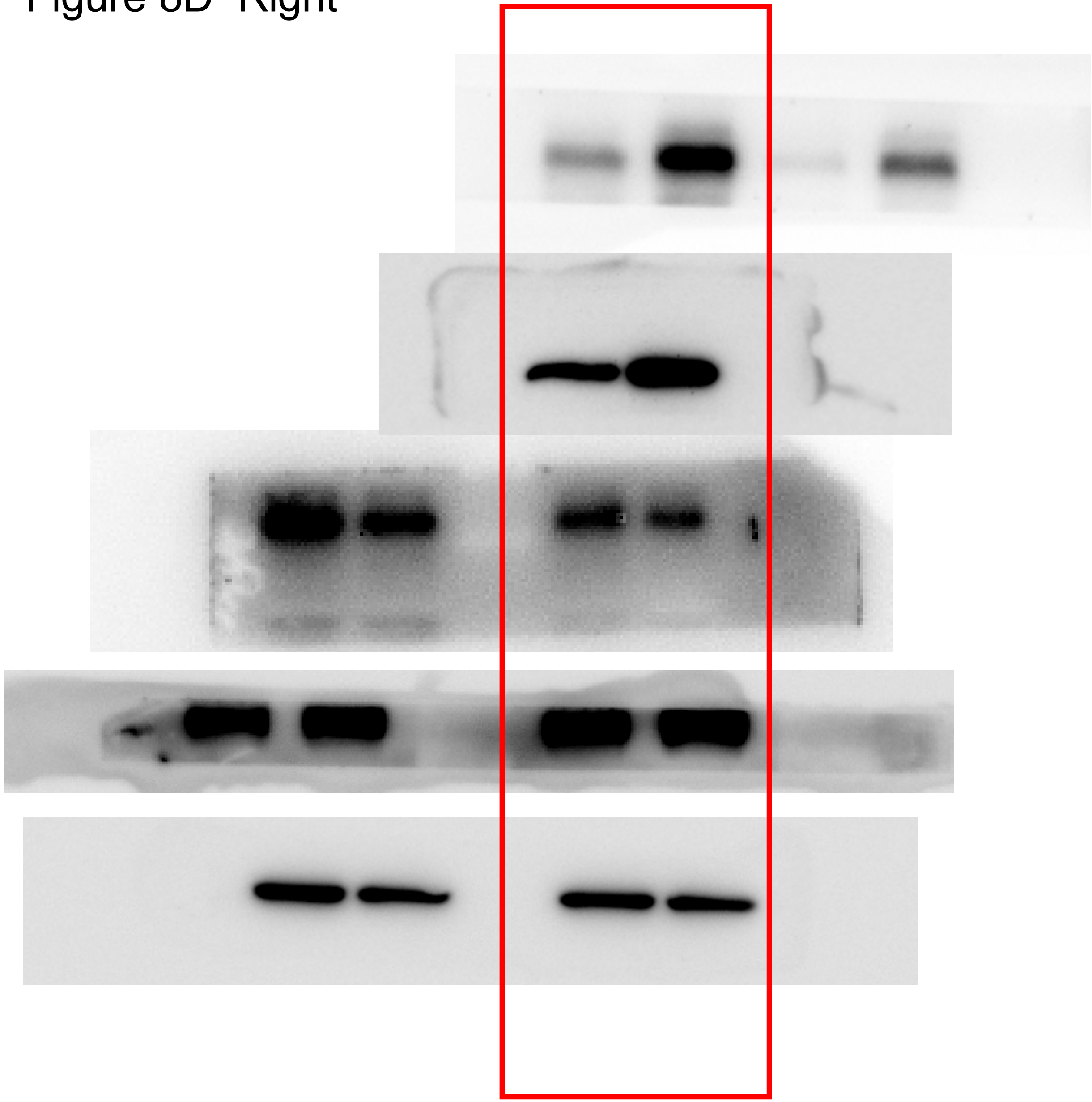

Figure 8F Left

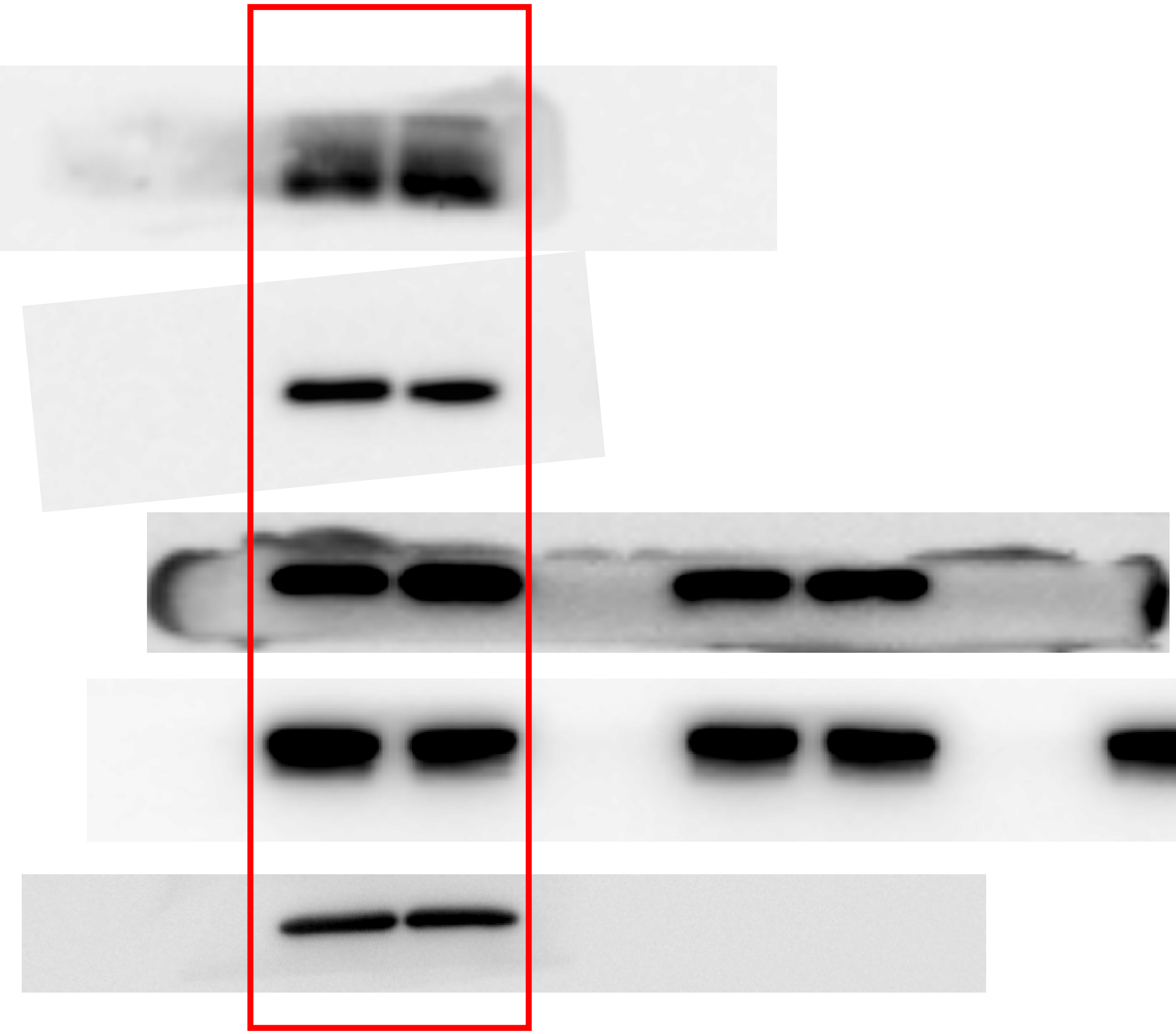

Figure 8F Right

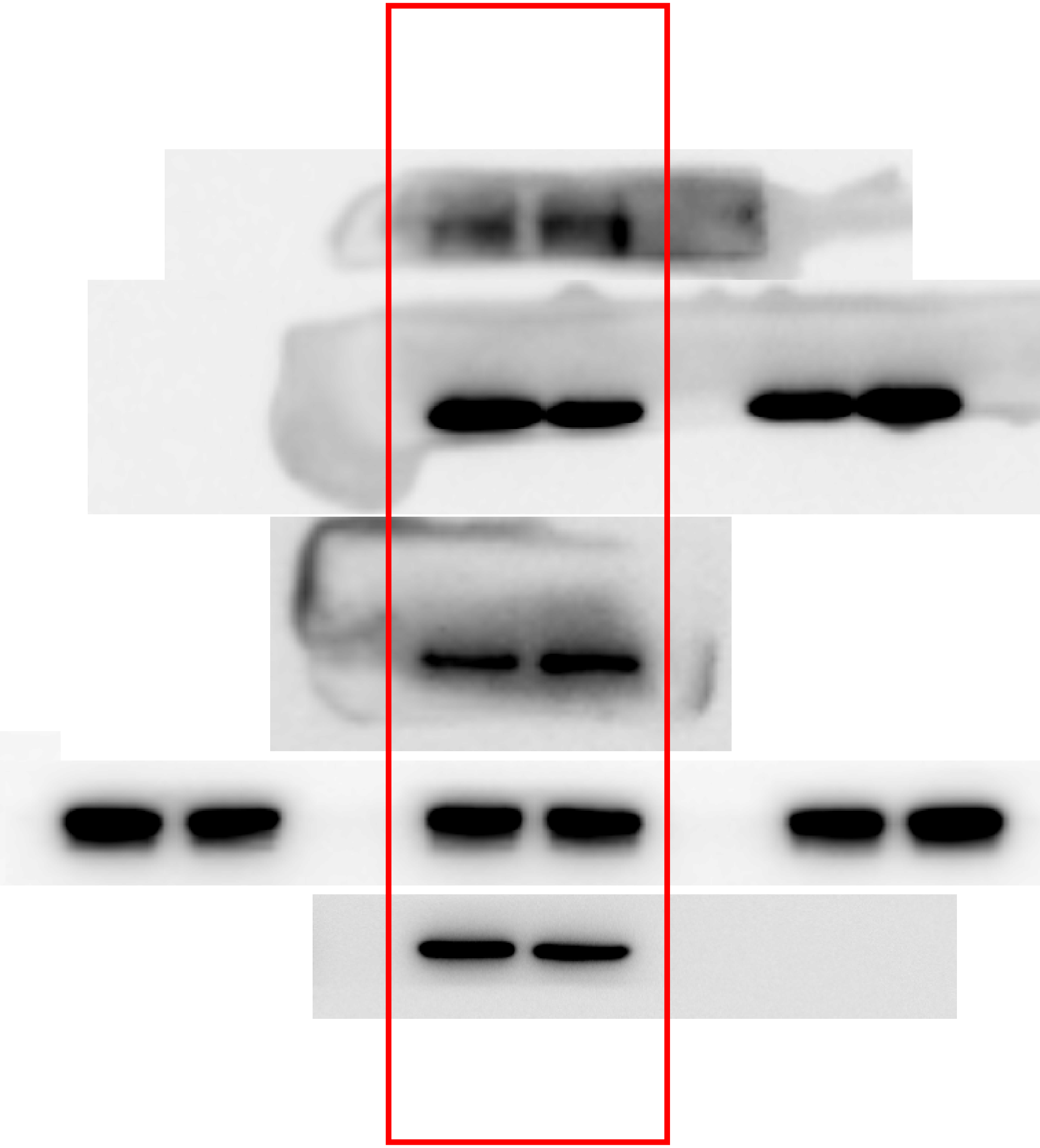

Figure 8H Left

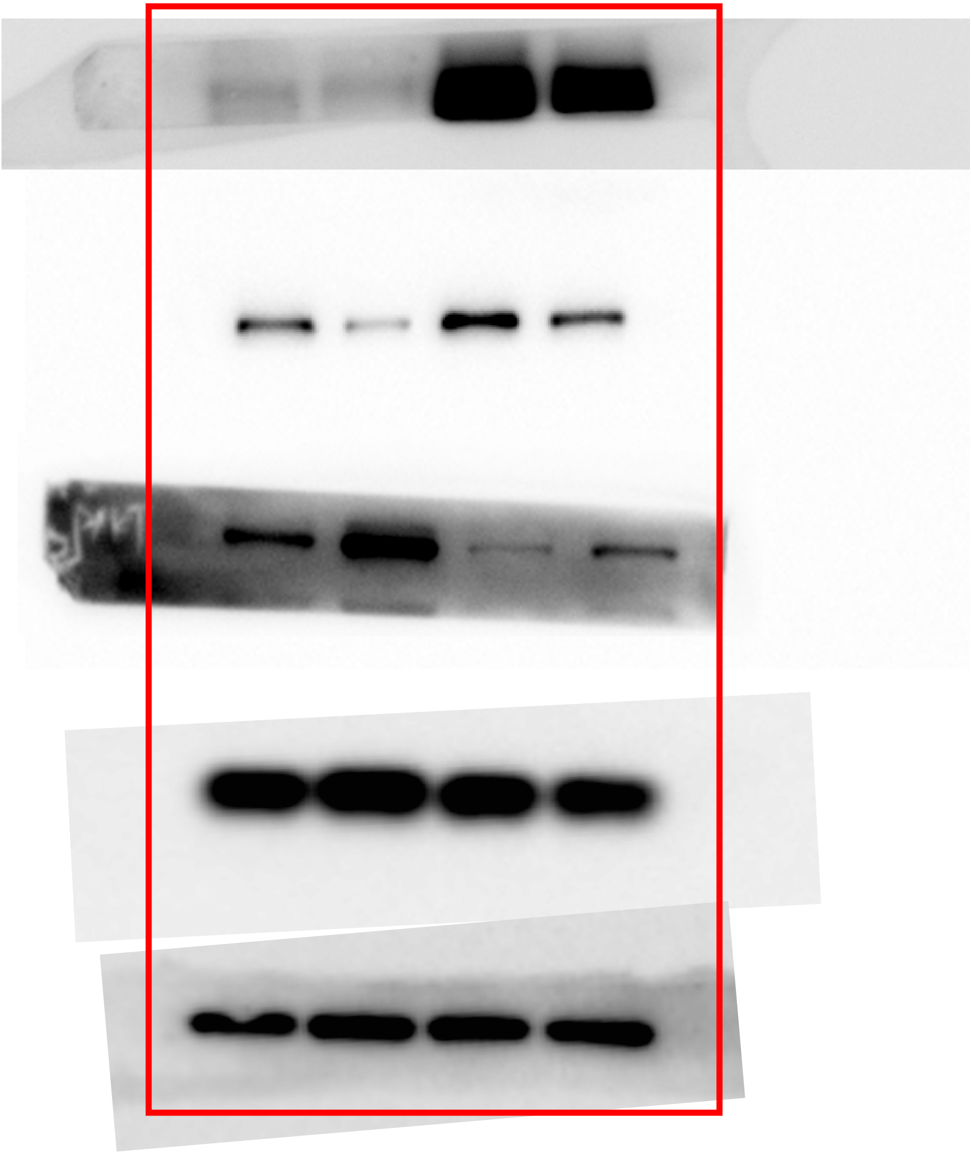

Figure 8H Right

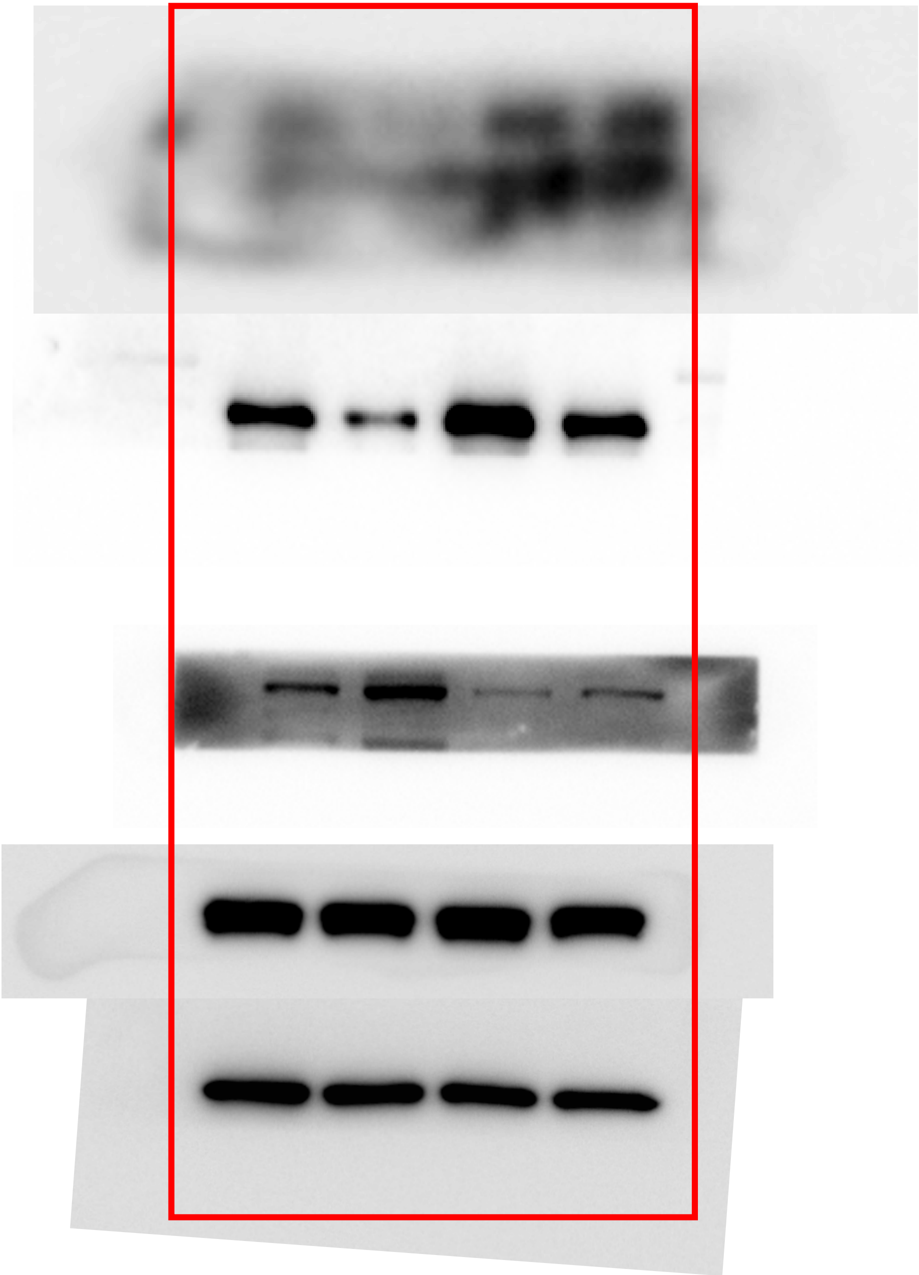

Figure 8J Left

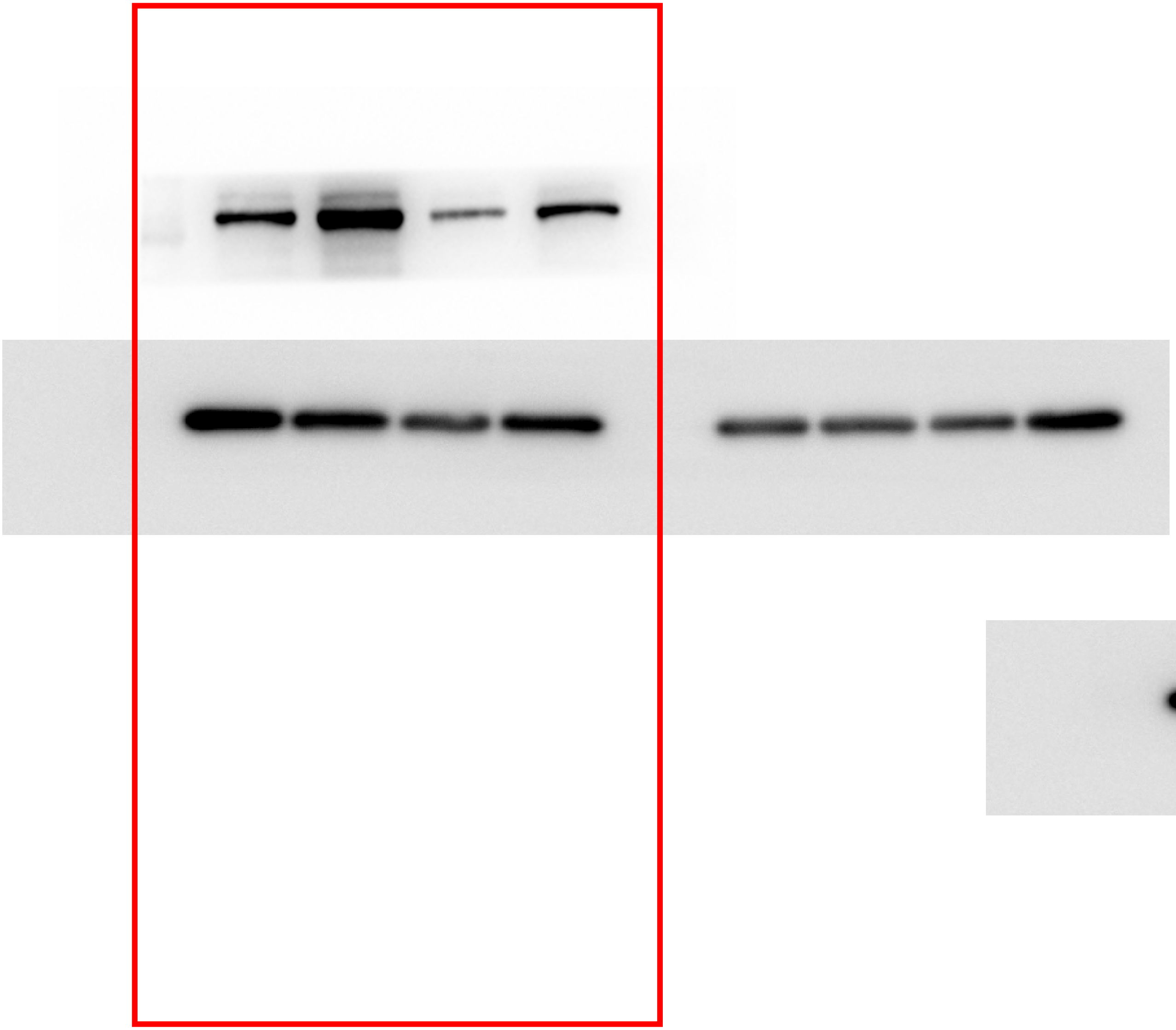

Figure 8J Right

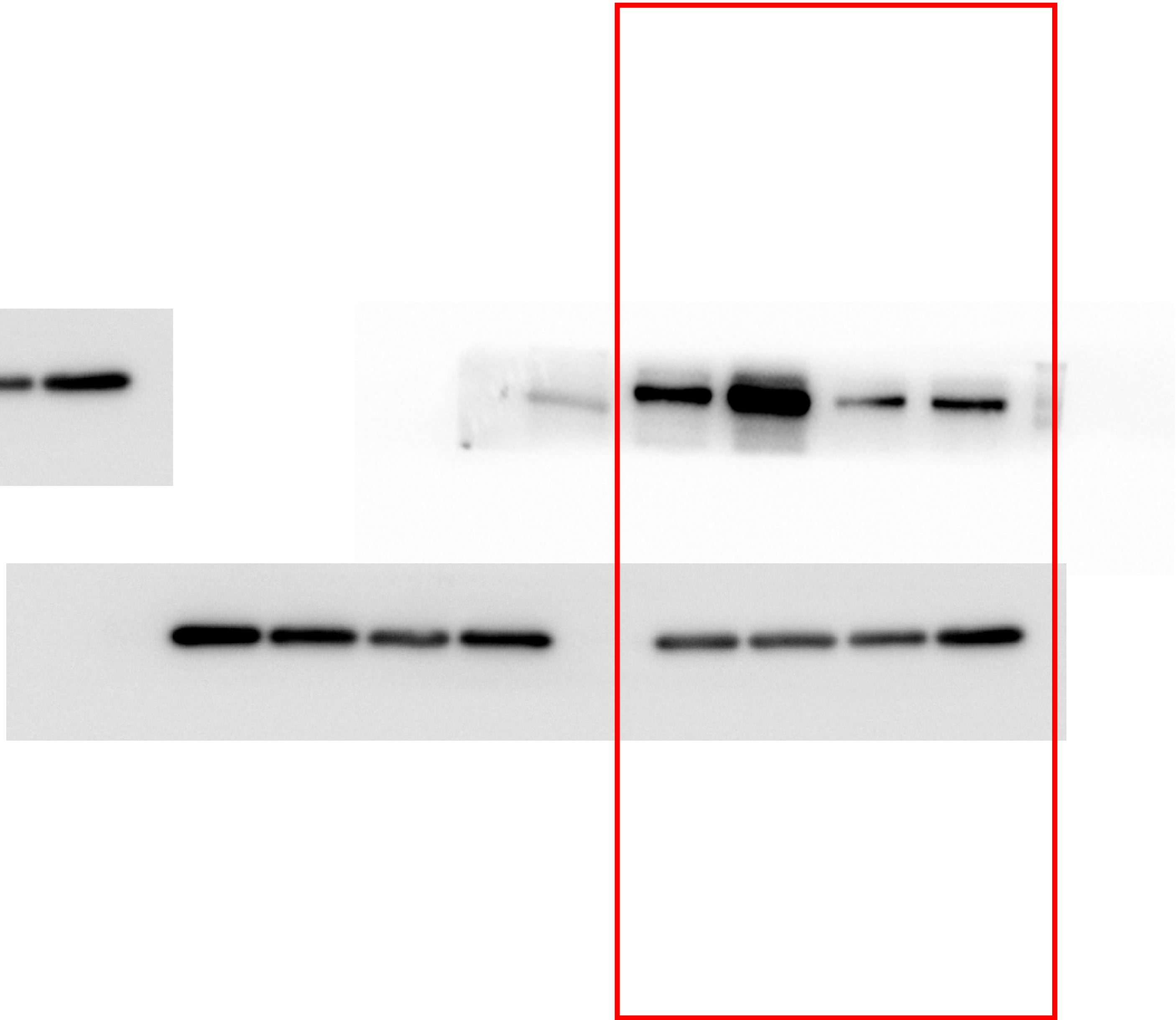

Supplement: Supplementary file 1 [file cancers-15-01261-s001.zip › File S1 Uncropped Western Blot images.pdf]
