# Supplementary material for: LncRNA LINC01094 Promotes Cells Proliferation and Metastasis through the PTEN/AKT Pathway by Targeting AZGP1 in Gastric Cancer
Source: Cancers (Basel). 2023 Feb 16;15(4):1261. doi: 10.3390/cancers15041261 (PMC9954187; doi:10.3390/cancers15041261)
Supplement: Supplementary file 1 [file cancers-15-01261-s001.zip › TableS1.pdf]

Table S1 Primers used in this study.

Gene	Primer sequence (5’-3’)
GAPDH	F: GGAGCGAGATCCCTCCAAAAT
	R: GGCTGTTGTCATACTTCTCATGG
LINC01094	F: GGCCACCAAGTCTGCAATTCTCC
	R: TCCCAGTGCTCCCTCTTCCTTTC
U1	F: GAAACTCGACTGCATAATTTGTGGTAG
	R: CTTGGCGTACAGTCTGTTTTTGAAACTC
AZGP1	F: GCGGAAATACCTGAAATACAGC
	R: AGTCCAGTGCACATCAATTTTC
PTEN	F: GACCAGAGACAAAAAGGGAGTA
	R: ACAAACTGAGGATTGCAAGTTC
